# Typical and anomalous pathways of surface-floating material in the Northern North Atlantic and Arctic Ocean

**DOI:** 10.1038/s41598-022-25008-5

**Published:** 2022-11-28

**Authors:** Agnieszka Herman, Jan Marcin Węsławski

**Affiliations:** Institute of Oceanology, Polish Academy of Sciences, 81-712 Sopot, Poland

**Keywords:** Ocean sciences, Physical oceanography

## Abstract

Surface waters of the oceans carry large amounts of material, including sediment grains, plankton organisms, and ice crystals, as well as pollutants, e.g., oil and plastic. Transport and spatio-temporal distribution of this material depend on its properties and on the dynamical processes in the ocean mixed layer—currents, waves, turbulence, and convective mixing—acting at a wide range of scales. Due to its importance for marine physics, biogeochemistry and ecology, substantial research efforts have been invested in recent years in observations and modelling of ocean material transport, especially in the context of marine plastic pollution. Nevertheless, many important questions remain unanswered. In this work, numerically simulated trajectories of surface-floating particles in the period 1993–2020 are used to analyse typical and anomalous transport pathways in the northern North Atlantic and the Arctic Ocean. Model validation is performed based on additional simulations of 387 buoy tracks from the International Arctic Buoy Programme in the years 2014–2020. The trajectories are computed based on surface currents from a hydrodynamic model and Stokes drift from a spectral wave model. It is shown that due to high amplitudes of Stokes drift (comparable with wind-induced currents in ice-free parts of the domain of study), combined with high directional variability, the drifting paths are substantially modified in ice-free regions, underlying the important role of wave-induced currents in surface material transport. A statistical analysis of $\sim 1.6\, 10^8$ trajectories reveals patterns of connections between nearshore locations in the domain of study, the associated drift times and path sinuosity. Seasonal variability of transport, which differs between the Arctic Ocean and the North Atlantic, is found for typical transport routes following the larger-scale circulation patterns. Crucially, in both sub-domains episodic, but very strong transport events between otherwise isolated locations occur, associated with anomalous atmospheric circulation and, arguably, providing ‘windows of opportunity’ for dispersal of various organisms to new locations. It is shown for two examples in the North Atlantic region that an unusual combination of atmospheric circulation indices explains the anomalous transport, thus providing a predictive tool for future events. In the Arctic, analogous phenomena are modified by the state of the sea ice cover.

## Introduction

Rapidly increasing levels of plastic pollution in the world oceans, extending to even the most remote locations, have motivated growing research efforts towards a better understanding of their associated processes, leading to a boom of studies on their physical, biological, ecological, as well as economic and industrial aspects^1–6^. At a more general level, plastic parts—from micrometre-scale particles up to meter-sized objects—are just one of many types of material transported by ocean currents. Therefore, studies on marine plastic can benefit from the substantial progress in observational, theoretical and numerical research on material transport in the ocean, including high-resolution modelling^7^, and vice versa, floating plastic particles, owing to the interest they attract, provide invaluable data^6,8,9^ for testing theories and validating models.

From a physical point of view, objects submerged in sea water—which throughout this paper will be broadly referred to as particles, or drifters—are characterised by their size, shape and density, which in turn determine the oceanic and, possibly, atmospheric drag forces that act on them, and eventually their response to forcing from the surrounding environment. Due to the very high variability of drifters’ properties, this response is extremely complex and sensitive to the details of the ocean mixed layer (OML) dynamics. To the first order, the fate of drifters in the OML depends on the sign and value of their characteristic vertical velocity relative to the surrounding water, i.e., their terminal velocity $w_t$. Four main types of drifters can be identified^7^, with $w_t<0$ (negatively buoyant drifters), $w_t=0$ (passive tracers), $w_t>0$ (positively buoyant drifters) and $w_t\rightarrow \infty$ (floaters, always staying at the ocean surface). The value of $w_t$ and the typical velocities associated with wind-induced stress, wave motion/Langmuir turbulence, and buoyancy^10^ determine particles’ drift speed and direction, their diffusion rates, vertical distribution in the water column and, as a result, their trajectories from source locations to their (final or temporary) removal from the system by beaching, sinking, decomposition, etc. In general, processes related to material transport in the OML can be divided into three groups: material production/release, the actual transport, and deposition/removal. In the case of plastic, our knowledge of spatiotemporal variability of its sources and sinks, and of the detailed mechanisms involved, is still far from satisfactory^1,2,6^. Moreover, in situ and remote-sensing observations of OML material transport are limited, and the very large range of spatial and temporal scales involved—from small-scale turbulence to basin-scale ocean circulation—makes generalizations based on existing data difficult or impossible. Consequently, numerical modelling remains the only tool allowing systematic analyses across scales.

In this study, as in several earlier ones^3,5,11–15^, Lagrangian (i.e., particle-tracking based) modelling is used. We concentrate on only one type of particles—passive floaters (as defined above)—and on their transport pathways, without addressing release and deposition processes. The area of study are polar and subpolar regions of the Northern Hemisphere, of particular interest for at least two reasons. First, several studies suggest the role of the Arctic as a sink of plastic and other marine pollution originating at lower latitudes, especially in densely populated and highly industrialized regions of North America and Europe^4,15–18^. Second, recent climate change and the associated changes of atmospheric and oceanic circulation, amplified in polar regions^19^, together with the associated negative trends of sea ice extent and thickness, very likely lead to modified transport routes of plastics and other material^20^. Taken together, modified pathways of plastic and other floaters to/within the warming Arctic mean new opportunities for numerous marine species, relying on those floaters as rafts, to migrate to new territories^21^.

**Table 1:** Coastal regions used in the study with their abbreviated names and number of particle release locations $N_r$.

No.	Region	Abbreviation	$N_r$
1	Alaska	ALA	122
2	Bear Island	BEI	12
3	Canadian Archipelago	CAA	146
4	Faroe Islands	FAR	23
5	Franz Josef Land	FJL	79
6	British Isles	GBR	181
7	Greenland, NE	GLN	145
8	Greenland, SE	GLS	119
9	Iceland	ICE	111
10	Jan Mayen	JAM	20
11	Norway	NOR	155
12	Russia (Europ.)	RUE	184
13	Siberia, east	SBE	178
14	Siberia, west	SBW	196
15	Shetland Islands	SHE	22
16	Svalbard	SVA	96

**Figure 1:**
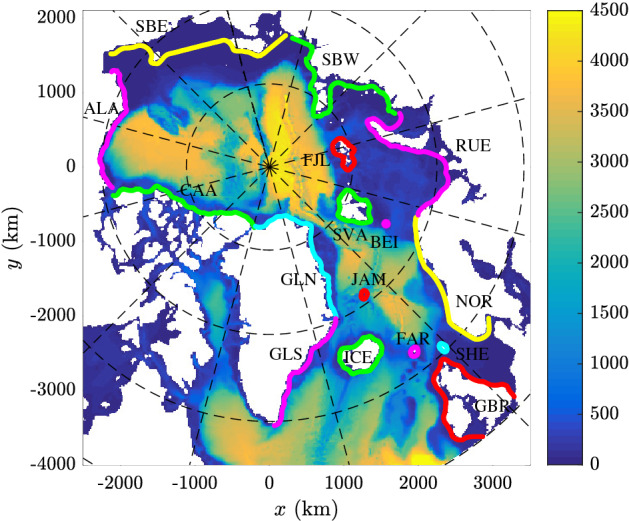
Map of the domain of study with particle release locations (see Table 1). The color shading shows depth in meters. (Map created with Matlab version 2016b, https://www.mathworks.com/).

In our simulations, 28 years (1993–2020) of ocean surface current and wind-wave data from the Arctic Ocean and the northern North Atlantic are used as input for Lagrangian modelling. The method is validated against observational data from the International Arctic Buoy Programme (IABP, https://iabp.apl.uw.edu/), as described in detail in Supplementary Note S2. In the main simulations, more than 160 millions trajectories are computed (see Methods), up to 3 years long, originating at 1789 near-shore locations grouped into 16 regions (Fig. 1 and Table 1). Based on those trajectories, probabilities of drifters originating at location A reaching location B are estimated, revealing the connection patterns between different coastal regions within the area of study. Probability density functions (pdfs) and mean values of drift duration and path sinuosity are computed for all connections. An analysis of time series of release and landing events for all A$\rightarrow$B pairs allows to identify seasonal and long-term variability of transport. Trajectories originating from two selected regions, the British Isles and Alaska, are used as representative drifter sources of the two main sub-domains of the area of study, the northern North Atlantic (predominantly ice-free; significant contribution of wave forcing) and the Arctic Ocean (sea-ice-covered throughout most of the year; contribution of wave forcing limited to ice-free areas and the marginal ice zone). In both cases, the transport connections can be divided into two broad groups: those that follow the typical paths associated with dominating, large-scale and regional circulation (e.g., from the British Isles to Norway and further towards the European part of Russia, or from Canadian Archipelago westwards towards Alaska and further to the East Siberian coast), and those associated with anomalous oceanic and/or atmospheric conditions, providing relatively fast, but short-lived paths between otherwise disconnected areas (e.g., from the British Isles or Norway to Iceland). Whereas the common feature of connections from the first group is their year-to-year persistence, usually with strong seasonal fluctuations, connections from the second group rely on specific, often exceptional forcing conditions, giving them an aperiodic, episodic character. As the examples analysed in this study demonstrate, those anomalous events are able to transport substantial amounts of material, thus contributing to spreading of pollutants, organisms and other material throughout the domain of study. Finally, the results of simulations with and without Stokes drift clearly demonstrate the crucial role wave-induced currents play in modifying surface material transport in ice-free areas, both in the open ocean and in the coastal regions, thus showing the limitations of studies based on wind-induced currents alone.

**Figure 2:**
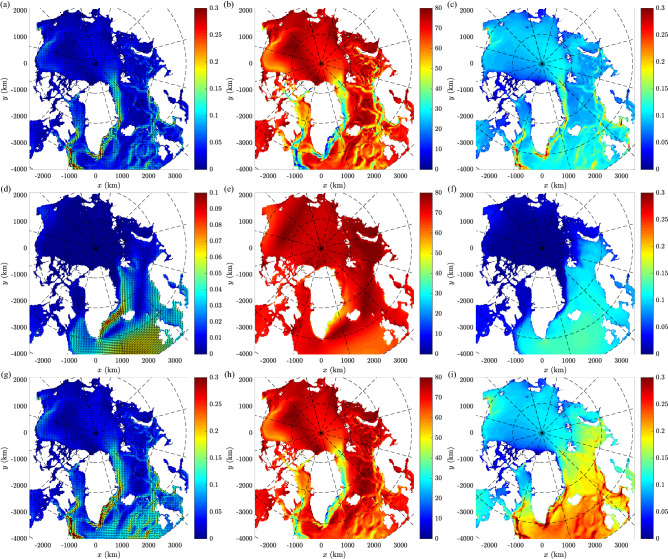
The 1993–2020 statistics of surface current velocities in the area of study: vector mean velocity (a,d,g; in m s$^{-1}$), standard deviation of current direction (b,e,h; in degr) and mean amplitude (**c**, **f**, **i**; in m s$^{-1}$) for $\mathbf {u}_c$ (**a**–**c**), $\mathbf {u}_S$ (**d**–**f**) and $\mathbf {u}=\mathbf {u}_c+\mathbf {u}_S$ (**g**–**i**). Note different color scales in (**a**, **g**) and (**d**). (Maps created with Matlab version 2016b, https://www.mathworks.com/).

**Figure 3:**
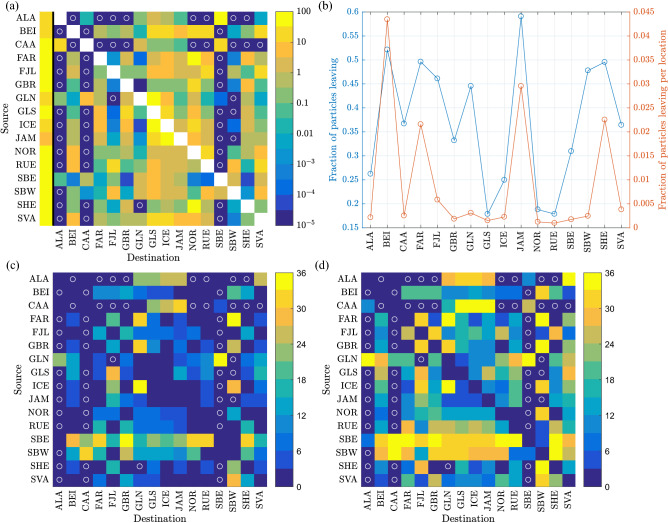
Statistics of transport connections between the 16 coastal regions in the area of study. (**a**): A connection matrix, showing the fraction of drifters (in percent) from a given source region (rows) reaching a given destination (columns). The first, unlabelled column represents particles that drifted out of the domain of study or are still afloat after 3 years. The sum of values in each row equals 100%. White circles mark cells with the value of zero. (**b**): Fraction of all particles released in a given region in the period 1993–2017 that escaped that region (left axis: in percent; right axis: divided by the number $N_r$ of release locations, see Table 1). (**c**, **d**): The minimum and median, respectively, time in months required for a particle from a given source region (rows) to reach a given destination (columns).

## Results

### Variability of surface currents in the area of study

Before investigating the particle trajectories and patterns of connections between different source and destination locations (Table 1 and Fig. 1), it is useful to analyze the variability of surface currents in the domain of study. The mean ocean and sea ice circulation in the Arctic Ocean and the North Atlantic is well known^19,22^; the data used as input in this study, averaged over the entire period 1993–2020, reproduce that circulation (Fig. 2a). At the large scale, the domain can be divided into two subregions, the northern North Atlantic with the European shelf seas (North Sea, Barents Sea), which remain mostly ice-free throughout the year, and the Arctic Ocean, where the presence of perennial or seasonal sea ice cover has a strong influence on the surface ocean dynamics. The dominant circulation features in the first region are as follows: the strong (southerly) flow along the east coast of Greenland and Newfoundland/Labrador (mean velocities up to 0.3 m s$^{-1}$), a cyclonic recirculation region in the area to the southwest of Iceland/east of southern Greenland (south of the Denmark Strait), and several branches of relatively stable north-easterly flow in the region west and north of the British Isles, further along the west and northwest coast of Norway and up to the west coast of Svalbard (mean velocities locally exceeding 0.1 m s$^{-1}$). Importantly, those main flow branches are largely independent of the local, instantaneous wind forcing, as they are part of the large- and regional-scale ocean circulation. In the remaining areas the vector-averaged currents are weak, at the level of a few centimetres per second. Crucially, however, that does not mean that the amplitudes of currents are similarly low in these areas (Fig. 2c); rather, their high short-term (synoptic scale) directional variability is reflected, associated with passing mesoscale weather systems. Over wide areas far from shore, the standard deviation of current directions (Fig. 2b) is close to 80$^\circ$, i.e., the value corresponding to the directionally uniform distribution (see “Methods”). Not surprisingly, that lack of directional stability is further enhanced by the Stokes drift (Fig. 2d,e). Apart from a few isolated locations at the coast of Greenland, the standard deviation of Stokes-drift directions is higher than 55$^\circ$ in all areas, and over most areas, it is higher than 70$^\circ$. Therefore, the vector-averaged Stokes velocities tend to be very low (Fig. 2d). However, their average amplitude over large parts of the North Atlantic is comparable or even larger than that of the wind-generated currents (Fig. 2c,f,i and Supplementary Fig. 1). Consequently, as mentioned in the introduction and as demonstrated in the next section, the contribution of the Stokes drift to the net drift of surface-floating material is substantial. Moreover, in near-shore and shallow areas waves tend to propagate towards the coast due to refraction and to increase their steepness due to shoaling, thus increasing the amplitude of the Stokes drift (note that those effects are not visible at the scale of maps in Fig. 2 and Supplementary Fig. 1, but they are present in the data). Therefore, in coastal areas Stokes drift facilitates the stranding of floating material, or at least its transport towards the inner coastal zone (see Supplementary Fig. 13c for an example).

Due to strong wave energy attenuation in sea ice (especially in the high-frequency range of the spectrum, strongly contributing to the net Stokes drift), the role of wave-induced flow in the ice-covered regions of the Arctic Ocean is limited, so that the total currents are close to those associated with sea ice drift within the Beaufort Gyre and the Transpolar Drift, with average amplitudes of 0.05–0.15 m s$^{-1}$. Still, the contribution of waves is noticeable in the seasonally ice-free areas of the southern Beaufort and Chukchi Seas, i.e., around coastal locations relevant for the subject of this study (Supplementary Fig. 13a,b).

**Figure 4:**
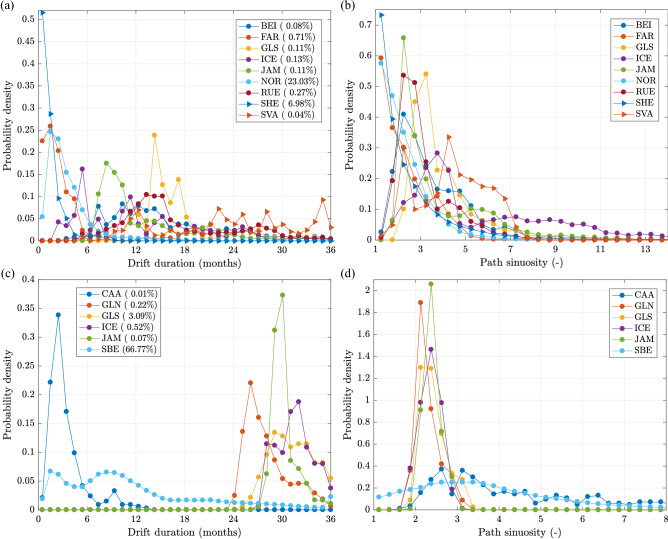
Probability distributions of drift duration (**a**,**c**) and path sinuosity (**b**, **d**) for trajectories originating at GBR (**a**, **b**) and ALA (**c**, **d**). The bin widths equal 1 month in (**a**, **c**), 0.5 in (**b**) and 0.25 in (**d**). Only those destination regions are shown, which are reached by at least 0.01% of all paths originating at a given source region (nine for GBR and six for ALA; values in the legend entries in panels **a**, **c**).

**Figure 5:**
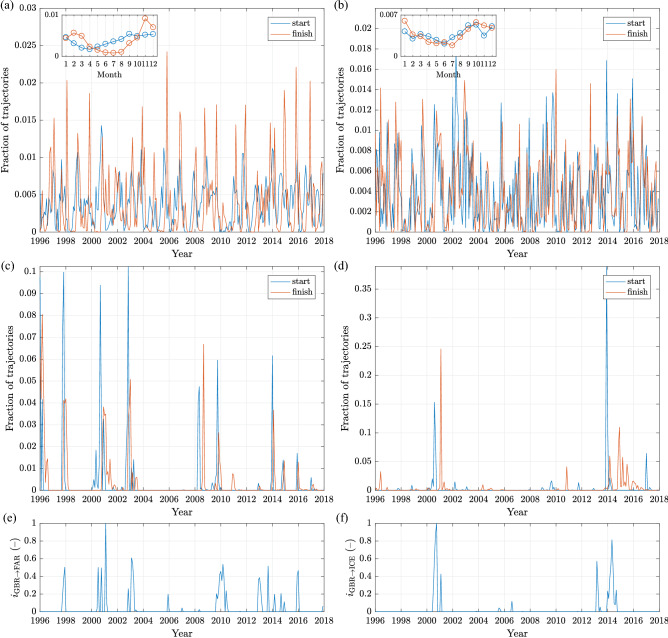
(**a**–**d**): Time series of total monthly release (blue) and landing (red) events from GBR to NOR (**a**), SHE (**b**), FAR (**c**) and ICE (**d**). (**e**,**f**): Time series (normalized with maximum values) of the atmospheric indices $i_{\mathrm {GBR}\rightarrow \mathrm {FAR}}(t)$ and $i_{\mathrm {GBR}\rightarrow \mathrm {ICE}}(t)$ defined in Eqs. (1)–(3). In all plots the period 1996–2017 is shown, for which a robust statistics of trajectories with duration 1 day–3 years can be computed. The labels at the *x*-axes are placed at the years’ begin. The insets in (**a**, **b**) show the mean seasonal cycles of release (blue) and landing (red) events.

### Patterns and variability of material transport

The large-scale, aggregate view of the surface material transport in the domain of study can be depicted in the form of a connection matrix, with rows/columns listing the source/destination regions, and cell values corresponding to the probability of particles travelling from source A to destination B (Fig. 3a). As for all source regions, a substantial (and, in some cases, a dominating) part of trajectories end on land within that same region after a few days of drift (Fig. 3b); hence, it is useful to exclude all A$\rightarrow$A-type trajectories from the connection matrix. In other words, for each source region we analyse the fate of those drifters that manage to escape that region and either reach one of the remaining 15 destinations or move out of the domain of study/stay adrift throughout the maximum time span considered (the first, unlabelled column in Fig. 3a). Notably, the fraction of drifters that escape their source region, normalized with the respective $N_r$ (proportional to the length of that region’s coastline), is roughly constant for all regions with an exception of small islands or groups of islands: BEI, FAR, JAM and SHE (red line in Fig. 3b). Not surprisingly, escaping is particularly difficult from elongated regions, especially those with relatively strong Stokes drift directed towards the shore (e.g., NOR, GLS or the west coast of GBR), as well as from regions with concave coastlines, trapping drifters within their semi-enclosed basins (e.g., RUE or SBW).

Considering the mean surface currents described earlier, the dominating transport directions are not surprising. For example, the south-east coast of Greenland (GLS) is the main destination for trajectories originating at GLN, JAM, ICE and SVA, i.e., source regions to the north, north-east and east of GLS, in the (average) upstream direction. Similarly, the Norwegian coast (NOR) is the main destination of floaters from GBR, SHE and FAR. Notably, due to the already mentioned relatively high speeds and directional stability of the major currents, these dominating pathways tend to be relatively fast (see the median and minimum travel times in Fig. 3c,d) and to have small sinuosity (defined as a ratio of the total path length to the shortest possible path, i.e., the straight-line distance between the start and end point).

As said, the connections described above are related to the main, long-term-average circulation. Arguably, the less frequent, episodic or anomalous paths are more interesting, as they provide means to shorten the usual, multi-stage trajectories and create connections between regions that otherwise are isolated from each other. The character of those anomalous connections is hard to decipher from the aggregate statistics in Fig. 3, but becomes clear in the in-depth analysis below, performed for two example source regions, one (GBR) representing the North Atlantic part of the domain of study, the other (ALA) the Arctic Ocean.

Undoubtedly, the British Isles (GBR) are a main source of anthropogenic drifters among the 16 regions analyzed (as our simulations suggest, it is also one of the main receivers, see Fig. 3a). On average, as this region is located close to continental Europe and to the external boundary of the domain of study (Fig. 1), the great majority (>80%) of trajectories originating in the southeasternmost and southwesternmost release locations around GBR are ‘lost’ to nearby destinations at the southern North Sea coast and through the boundary, respectively. Of the remaining ones, the majority reach Norway (NOR; $\sim$23%), the Shetland Islands (SHE; $\sim$7%) and the Faroes (FAR; $\sim$0.7%). Overall, there are nine destination regions that receive at least 0.01% of all particles leaving GBR (Figs. 3a and 4a,b), enough for computing robust statistical measures of those paths. The remaining regions receive no (ALA, CAA, SBE) or only very few isolated particles (FJL, GLN, SBW).

As might be expected due to their spatial proximity, the typical drift duration from GBR to NOR, SHE and FAR is short (Fig. 3c,d), peaking at 1–2 months and, especially for SHE, rarely longer than 5–6 months (Fig. 4a). The corresponding pdfs of sinuosity are nearly exponential (Fig. 4b). However, the character of each of these three drift connections is very different, as can be seen from the time series of monthly particle departure and arrival events (Fig. 5a–c). In the case of NOR, the main receiver of material from GBR, transport takes place with comparable intensity every year, with high-amplitude, repeatable seasonal fluctuations, which are particularly strong in the case of landing times: all high peaks of landing visible in the time series in Fig. 5a occur between October and March, with the largest values in November and December. Although seasonal variability is also present in the GBR$\rightarrow$SHE case (Fig. 5b), due to a short distance (note that particles reaching SHE originate mostly in the northern part of GBR, i.e., in Scotland and Northern Ireland) the seasonal variability is overlapped by synoptic-scale weather patterns. The short distance and thus drift time makes successful GBR$\rightarrow$SHE transport possible even if duration of favourable conditions is relatively short. This is not the case for GBR$\rightarrow$FAR and, even more so, GBR$\rightarrow$ICE transport. The straight-line distance GBR$\rightarrow$FAR, depending on the exact release and landing location, averages 550 km, with a minimum of 330 km; for GBR$\rightarrow$ICE, the values are $\sim$1100 km and 800 km, respectively. Thus, with usual drift speeds of, say, 0.1–0.2 m s$^{-1}$, 20–40 days (respectively 45–90 days) are necessary to cover the smallest distance from GBR to FAR (respectively from GBR to ICE). In both cases, the net drift direction (relative to the ocean floor) has to be roughly perpendicular to the mean northeasterly currents in that region (Fig. 2), i.e., apart from a velocity component perpendicular to the mean flow, a component opposing that flow is necessary to prevent the floaters from moving too far east before they drift sufficiently far north. To make that possible, anomalous wave/current forcing, and thus anomalous atmospheric circulation patterns, are necessary, either present over extended periods of time or causing exceptionally high drift speeds in north-westerly directions in the area in question. Accordingly, the GBR$\rightarrow$FAR paths tend to have low sinuosity, often close to the minimum value of 1, corresponding to a straight line, i.e, the drift is fast and unidirectional. In the case of GBR$\rightarrow$ICE, the distribution of path sinuosity has a broad peak in the range 2.5–5.0 and a wide tail, but, remarkably, the paths from the two largest GBR$\rightarrow$ICE transport events, clearly seen in Fig. 5d, are characterized by well-below-average sinuosity values.

Over the whole 28-year-long period of study, more than one particle leaving GBR in a thousand (0.13%) landed on ICE. This is a substantial number, especially if one considers that almost all of those particles were transported within just two events (Fig. 5d), one in 2000–2001 (with release peak in August 2000 and landing peak in February 2001) and one in 2013–2015 (with the release peak in December 2013 and landing distributed over several sub-events in late 2014 and throughout 2015). The scale and exceptional character of those two events indicates highly anomalous wind forcing. Indeed, their occurrence coincides with a rare combination of those atmospheric circulation indices that have their associated spatial patterns active over the relevant part of the North Atlantic: the North Atlantic Oscillation (NAO), East Atlantic (EA), East Atlantic/Western Russia (EA/WR) and Scandinavian (SCAND) patterns (see “Methods” and Supplementary Note S3). For an anomalous wind forcing in the northwesterly direction, positive phases of EA and SCAND and a negative phase of EA/WR are favourable, together with a small amplitude of NAO, i.e., weak overall northeasterly flow. Let us denote the time series of those indices, smoothed with a moving-average window of 9 months, with $i_\mathrm {NAO}(t)$, $i_\mathrm {EA}(t)$, $i_\mathrm {SCAND}(t)$ and $i_\mathrm {EAWR}(t)$, respectively, and define a new index, $i_{\mathrm {GBR}\rightarrow \mathrm {ICE}}(t)$, as a product of four terms:

1 $$\begin{aligned} i_{\mathrm {GBR}\rightarrow \mathrm {ICE}}(t) = \max \{i_\mathrm {EA}(t),0\} \max \{i_\mathrm {SCAND}(t),0\} |\min \{i_\mathrm {EAWR}(t),0\}|\alpha _\mathrm {NAO}(t), \end{aligned}$$

where:

2 $$\begin{aligned} \alpha _\mathrm {NAO}(t) = \left\{ \begin{array}{ccc} 1 &{} \mathrm {if} &{} |i_\mathrm {NAO}(t)|<0.25, \\ 0 &{} \mathrm {if} &{} |i_\mathrm {NAO}(t)|\ge 0.25, \end{array}\right. \end{aligned}$$

The two periods of non-zero values of $i_{\mathrm {GBR}\rightarrow \mathrm {ICE}}$ correspond well to the maxima of GBR$\rightarrow$ICE transport (Fig. 5d,f). (Although it is impossible to verify that prediction based on the available data, the values of $i_{\mathrm {GBR}\rightarrow \mathrm {ICE}}$ computed for the period 1950–2020 suggest very strong GBR$\rightarrow$ICE transport events in 1952 and 1988, and weaker ones in 1961 and 1972.) For GBR$\rightarrow$FAR, the best combination of the basic indices is found as:

3 $$\begin{aligned} i_{\mathrm {GBR}\rightarrow \mathrm {FAR}}(t) = \max \{i_\mathrm {EA}(t),0\} \max \{i_\mathrm {SCAND}(t),0\} |\min \{\nabla i_\mathrm {EAWR}(t),0\}|, \end{aligned}$$

i.e., it is independent of NAO and includes the gradient of EA/WR instead of its values (that is, conducive for GBR$\rightarrow$ICE transport are periods of decreasing EA/WR). As can be seen in Fig. 5c–f, the agreement is not perfect—and hard to verify statistically due to the small number of events. Nevertheless, the indices (1)–(3) seem to provide a good rough guess of the likelihood of the GBR$\rightarrow$FAR and GBR$\rightarrow$ICE transports to occur. It is important to stress that in both cases it is the combination of the basic indices that provides a good predictor of transport and not any of these indices alone: none of EA, EA/WR, SCAND had exceptionally high amplitude during the events in question; and omitting any of the terms in Eqs. (1) and (3) spoils the performance of $i_{\mathrm {GBR}\rightarrow \mathrm {ICE}}$ and $i_{\mathrm {GBR}\rightarrow \mathrm {FAR}}$. In general, as shown in Supplementary Note S3, the correlation between the individual atmospheric indices and time series of monthly release and landing events (like those in Fig. 5a–d) is low and rarely exceeds ±0.2. Non-negligible values tend to occur for the ‘typical’ routes, e.g., to NOR from GBR, SHE and FAR (positive correlation with NAO and negative with SCAND). Otherwise, as in the example of GBR$\rightarrow$ICE discussed above, a combination of several factors is necessary.

**Figure 6:**
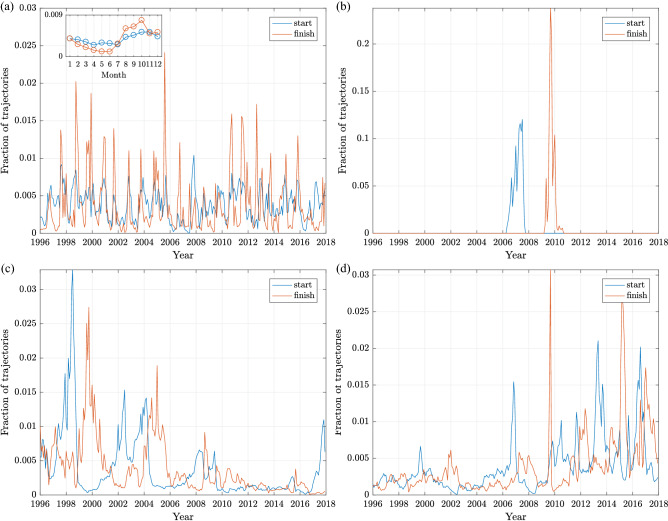
As in Fig. 5, but for transport ALA$\rightarrow$SBE (**a**), ALA$\rightarrow$GLS (**b**), GLN$\rightarrow$CAA (**c**) and CAA$\rightarrow$GLN (**d**).

The hitherto analysis concentrated on locations in the North Atlantic sector of the domain of study. The trajectories originating at ALA provide a good example of transport patterns in the second sub-domain, the Arctic Ocean. There are six destinations that receive floating material from ALA in significant quantities (Figs. 3 and 4c,d): two neighbouring ones, CAA to the east (i.e., upwind/upstream) and SBE to the west (i.e., downwind/downstream), and four remote ones, GLN, GLS, ICE and JAM. As on average the drift velocities in the Arctic are very low (Fig. 2), all features of trajectories leading to those two groups of destinations are markedly different. Connections ALA$\rightarrow$CAA and, especially, ALA$\rightarrow$SBE are active every year and exhibit a typical seasonal variability, although a different one than that observed in lower-latitude, ice-free regions: here, the maxima of landing frequency occur between August and December, with the highest values in August–October (Fig. 6a), that is, during the Arctic summer/autumn, when the nearshore areas of the Beaufort and Chukchi Seas are free of ice or covered with low-concentration, drifting ice pack. Usually, the drift duration to SBE and CAA is a few months and the paths often have relatively high sinuosity (Fig. 4c,d), reflecting the varying wind conditions (see Supplementary Fig 9 for selected path examples).

**Figure 7:**
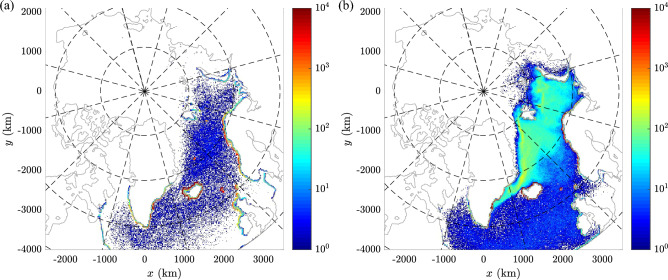
Maps of the spatial density of final positions of particles released from ICE in the period 1993–2017 (in number of particles per grid cell, i.e., $\sim$156 km$^2$) in simulations with (**a**) and without (**b**) Stokes drift. The color scale is logarithmic, grid cells with no particles are white. (Maps created with Matlab version 2016b, https://www.mathworks.com/).

In order for the drifters to reach Greenland or Iceland, however, they have to not only cover large distances, first within the Beaufort Gyre and then the Transpolar Drift, but also encounter conditions favorable for crossing the Transpolar Drift in the easterly direction; otherwise, they return towards the North American coast within the eastern branch of the gyre. As can be seen in Fig. 6b with the example of the ALA$\rightarrow$GLS connection (time series for GLN, ICE and JAM are similar), there was only one ‘mega-event’ in the whole study period when the drift successfully completed within the time limit of 3 years: a very large number of drifters that left ALA between early 2006 and late 2007 reached GLS from throughout 2009 until early 2010, i.e., during the very strong negative phase of the Arctic Oscillation (AO) index (Supplementary Figs. 17 and 19e). The timing of that event corresponds to anomalous intensification of the Beaufort Gyre in the period 2007–2010, manifested in its increased doming^23^ and amplification in ice-drift curl^24^, combined with anomalies in the speed and orientation of the Transpolar Drift (TPD)^20,25^. In particular, the year 2007 was characterized by the strongest cross-TPD sea ice transport in the whole period 1979–2015^25^. Remarkably, the period 2007–2009 seems also to mark a period of reversal in material transport between the two neighbouring regions of GLN and CAA (Fig. 6c,d), with transport GLN$\rightarrow$CAA dominating in the first and CAA$\rightarrow$GLN in the second part of the analysis period. Again, climate-change driven changes of the sea ice cover are likely responsible for that rapid change of drift pattern in that area.

Overall, the results of this study demonstrate the very clear separation between the two sub-domains of the area of study. The maps showing spatial density of the final positions of all trajectories originating from a given source region (Supplementary Note S4) can be broadly divided into two groups. Drifters originating in the western part of GLN, as well as in CAA, ALA and SBE stay within the Arctic Ocean or leave it through the Bering Strait and, especially, in a wide southerly stream along the east coast of Greenland. Crucially, none of these drifters reach any of the European destinations between GBR, NOR and RUE, and only a few (from SBE) reach SBW. Analogously, drifters originating in the northern North Atlantic only sporadically enter the Arctic Ocean. Notably, this is true not only for sources in Iceland, British Isles and continental Europe, but also for the European Arctic islands: SVA, BEI, FJL and islands belonging to RUE. The only exception is SBW: although its main receivers are located in the European Arctic, trajectories from this source regularly enter the Arctic Ocean (however, they only sporadically reach the coast there; rather, they leave again with the East Greenland current).

## Discussion

One of the main findings of this study is the existence of episodic transport events that connect otherwise separated coastal regions and are capable of transporting substantial amounts of floating material between those regions. As illustrated with the examples of GBR$\rightarrow$ICE and ALA$\rightarrow$GLS connections, these events are associated with anomalous atmospheric circulation and, in the case of the sea-ice covered part of the Arctic Ocean, can be amplified by favourable sea ice conditions. Such events are able to transport all kinds of material, both natural and anthropogenic, along atypical routes; in particular, they provide ‘windows of opportunity’ for marine organisms to disperse and colonize new locations. Episodic dispersals related to anomalous (in terms of direction and/or speed) advection are known from different regions of the world ocean, including Antarctica^26^, the north-east Pacific^27^, the tropical Atlantic^28^, and Svalbard^29^. The rapid connections are particularly relevant for benthic organisms, especially those belonging to the coastal fauna. The survival of their planktonic larvae is typically very short and does not exceed a few weeks^30^. Therefore, to reach new, remote locations they either require exceptionally fast drift, preferably during the summer season^29^, or natural or man-made objects that adult organisms can use as rafts^21,31^. Shorter drift substantially increases survival chances of those organisms, making anomalous transport events very important for the biology and ecology of the affected regions.

When interpreting the results of this study, it is important to remember their range of validity and limitations following from the data used as input and from the assumptions underlying the computation of trajectories. As mentioned several times, this analysis is limited to surface-floating particles, with $w_t\rightarrow \infty$. The paths of objects protruding from the water and therefore subject to windage might be substantially different^1^. The same is true for drifters with $0<w_t<\infty$, the transport of which is a net effect of the complex, three-dimensional OML dynamics, shaped by combined Ekman–Stokes flow, as well as buoyancy^1,7,10^. It has been shown for several types of particles, e.g. oil spills^32^ and frazil ice^33^, that the spatio-temporal three-dimensional patterns of particle concentration, as well as the overall transport speed and direction, are very sensitive to particle size. Another issue worth mentioning is related to the beaching and de-beaching processes. As described in “Methods”, it is assumed that trajectories reaching the coastline terminate there. In reality, a certain fraction of objects washed onshore is washed off again; in principle, their multi-segment trajectories could be recreated by combining several trajectories computed in this study, with common end and start locations. For elongated coastal regions, e.g. NOR, this would result in ‘leap-frog’ paths with several stranding events, which, one can speculate, is the actual mode of transport of some types of floating material there.

A very important aspect of the results presented in this paper is related to the role the Stokes drift plays in shaping the patterns of material transport in the domain of study, especially in its ice-free North Atlantic sector. That role can be reliably assessed here, as the surface Stokes currents are computed from full wave energy spectra (see “Methods”), as opposed to earlier approaches that relied on integral wave parameters^1^. Notably, the surface Stokes drift for monochromatic deep-water waves is proportional to $\omega ^3a^2$ (where $\omega$ and *a* denote wave angular frequency and amplitude, respectively), i.e., short, relatively steep waves from the high-frequency part of the wave energy spectrum contribute more strongly to the net drift than long waves around the peak of the spectrum^34^. This fact explains the high amplitudes of the Stokes drift discussed earlier (compare Fig. 2d–f and Supplementary Fig. S2, which shows analogous statistics for Stokes drift velocities computed from the significant wave height, peak wave period, and mean wave direction). The results of validation against the AIBP buoy tracks from open-water areas show better performance of the model when the Stokes drift is taken into account (Supplementary Figs. 7c,d and 13a,b). The role of Stokes drift is also clearly visible in the example in Fig. 7, showing final positions of particles originating at ICE, computed with and without Stokes drift. In the second case, confirming results of earlier studies^5,26,35,36^, a substantial part of drifters remains afloat over very long periods of time, as they do not get stranded even when they pass very close to the shore (see also Supplementary Fig. 13c). Effectively, all features of trajectories are very different. This shows that results of models that do not take Stoke drift into account, or that estimate it based on the integral wave parameters, should be treated with caution. Importantly as well, the contribution of the wave-induced drift to the total velocity at the sea surface is likely even larger than suggested by the data used here. First, no drift associated with breaking waves is included. Second, it is well known from observations and LES models that in well-developed Langmuir turbulence, the horizontal drift velocity is largest within the surface convergence zones (windrows), where it might reach 150% of the average^37^—and the type of material considered here very likely tends to accumulate in those zones. And finally, yet another mechanism has been described recently^38^, leading to enhanced drift speeds of buoyant objects of large sizes.

The fact that waves substantially contribute to the surface material transport in the ocean is important from yet another perspective—that of the recent climate change. Increased wave activity related to stronger and/or more frequent storms, as well as shifting storm tracks, have been observed in the recent past in different regions of the area of study^39–41^. In the Arctic, negative trends of sea ice extent are associated with increasing wind fetch and higher waves, especially in the summer, i.e., the season of intense Arctic storms. Though beyond the scope of this study, we might speculate that waves in ice-free areas along the North American coast contribute to the shift of transport in the CAA–GLN area visible in Fig. 6c,d. In any case, whereas mean and anomalous transport in the northern North Atlantic can be explained with changes atmospheric forcing, analogous transport in the Arctic is driven by both wind and changes in the state of the sea ice cover^23,24^.

## Methods

The analysis in this work is based on two data sources, available through the Copernicus Marine Service, part of the European Union’s Earth Observation Programme (https://marine.copernicus.eu/): the “Arctic Ocean Physics Reanalysis” (https://resources.marine.copernicus.eu/product-detail/ARCTIC_MULTIYEAR_PHY_002_003/), and the “Arctic Ocean Wave Hindcast” (https://resources.marine.copernicus.eu/product-detail/ARCTIC_MULTIYEAR_WAV_002_013/). Both datasets contain results of numerical models run operationally at the Norwegian Meteorological Institute: the Hybrid Coordinate Ocean Model (HYCOM), using the coupled ensemble data assimilation system TOPAZ4b and coupled with a sea ice model, and the WAve Model (WAM) version 4.7.0. Both models are forced with ERA5 atmospheric data. Among variables assimilated in HYCOM are satellite-derived sea ice drift velocities from CERSAT, Ifremer. The products used in this work are: daily-mean velocity components of the surface currents, $\mathbf {u}_c=(u_{c,1},u_{c,2})$, available on a polar stereographic grid with spatial resolution of 12.5 km; and hourly, instantaneous components of the surface Stokes velocity, $\mathbf {u}_S=(u_{S,1},u_{S,2})$, available on a polar stereographic grid with spatial resolution of 3 km. In this study, data from the years 1993–2020 are used from a rectangular region common to the two datasets, i.e., covering the entire Arctic Ocean and the North Atlantic north of $\sim$50$^\circ$N (Fig. 1). Importantly, the HYCOM results include effects of winds and tides. The surface Stokes drift is computed from the full wave energy spectra, as described in the documentation of the ECMWF Wave Model (https://www.ecmwf.int/file/285503/). At the data preparation stage, the Stokes velocity components are daily-averaged and linearly interpolated onto the HYCOM grid. The vector sum $\mathbf {u}=\mathbf {u}_c+\mathbf {u}_S$ is then used as an input to compute particle trajectories. Wherever sea ice is present, its velocity $\mathbf {u}_i$ is used for $\mathbf {u}_c$.

For the sake of clarity in the analysis and presentation of the particle drift data, the coastal zones in the domain of study are divided into 16 regions, labelled with three-letter codes (Fig. 1 and Table 1). Along the coastlines of each region, in the distance of $\sim$30 km from shore and spaced at $\sim$15 km, particle release locations are defined (see $N_r$ in Table 1; there are $N_{r,\mathrm {tot}}=1789$ locations in total). Each release location is in fact a square region with side length of 5 km centred at a given coordinate. From each location, particles are released once per day, with random positions within their respective squares, and allowed to drift along stochastic trajectories $\mathbf {x}(t)=(x_1(t),x_2(t))$ computed from a discretized Fokker–Planck equation in the form:

4 $$\begin{aligned} x_k(t+\Delta t)=x_k(t)+u_k(t)\Delta t+R(t)\sqrt{2K_h|\Delta t|}, \quad \hbox {for}\quad k=1,2. \end{aligned}$$

where *t* denotes time, $\Delta t$ is the integration time step (positive or negative for forward or backward trajectories, respectively), *R* is a random number drawn from a $[-1,1]$ interval, and $K_h$ denotes the horizontal diffusion coefficient^3,42^.

There are two main sources of uncertainties in the results of this study. First, there are errors associated with the results of the HYCOM and WAM models. Validation reports of both models, based on satellite data and in situ observations, are available with their documentation at the links provided above. In order to assess the suitability of that data for our purposes, as well as to test the method itself, we performed additional simulations for 387 buoy tracks from the International Arctic Buoy Programme (IABP; note that IABP tracks are not assimilated in HYCOM, i.e., are suitable for validation as an independent data source). The results of validation, described in Supplementary Note S2, show that the simulated trajectories reproduce the observed ones with sufficient accuracy; they also confirm that the model version with Stokes drift performs better in regions without sea ice or with low ice concentration. The second source of uncertainties mentioned above is related to the (generally unknown) value of $K_h$ in equation (4). In this study, it is assumed constant and is estimated from^3,11^:

5 $$\begin{aligned} K_h = K_{h,0}(l/l_0)^{4/3}, \end{aligned}$$

where $K_{h,0}=1$ m$^2\,$s$^{-1}$, $l_0=1\, 10^3$ m, and *l* is the spatial resolution of the data. With $l=12.5\, 10^3$ m, we have $K_h=29$ m$^2\,$s$^{-1}$. This value, together with $\Delta t=24$ hours, is used in the set of simulations analysed in the main text of this paper. Additionally, several sensitivity studies have been performed, with $K_{h,a}=10K_h$, $K_{h,b}=0.1K_h$, as well as $\Delta t_n=\Delta t/n$ with $n=8$ (in this case, particle positions were updated *n* times per day, but the current maps were kept constant throughout a whole day). The differences between results with $n=1$ and $n=8$ turned out to be insignificant and are not discussed further. The results obtained with different $K_h$ are described in the Supplementary Note S1. They show that, although the range 2.9–290 m$^2\,$s$^{-1}$ is much wider than $K_h$ values actually occurring in the ocean^43^, the main conclusions from this study remain unaffected. (It is noteworthy that the buoy data used for validation cannot be used to constrain $K_h$, as there are no groups of buoys released at the same time from the same location that would enable a comparison between the modelled and observed track dispersion. Therefore, the validation and the sensitivity analysis in Supplementary Notes S1 and S2 are treated as separate problems.)

In all simulations, the maximum allowed duration of drift $t_\mathrm {max}$ equalled 3 years. Thus, for forward trajectories considered in this work, the allowed release dates are between 01.Jan.1993 and 01.Jan.2018, i.e., cover $N_d=9132$ days. With $N_p=10$ particles released from each location, this results in the total of $N_{r,\mathrm {tot}}N_pN_d\approx 163\, 10^6$ trajectories.

During their drift, the particles might leave the domain through its southern boundary in the North Atlantic, reach one of the coastal regions (including the one from which they had been released), or stay adrift throughout their whole life period of 3 years. No de-beaching is used, i.e., trajectories that reach land are terminated there. This makes the interpretation of the results easier and removes from the analysis poorly constrained parameters (for instance, the probability of the beached particles to get washed again to the sea, which depends on the local morphology of the coastal zone, its surface type and several other highly variable factors that cannot be reliably estimated, especially at spatial scales considered here). Crucially, as follows from the description above, no information on the absolute or relative amounts of particles that in reality are released from the different regions is taken into account. The questions this study aims to answer are: if a floating object leaves a nearshore location A (at a particular time instance or within a specified time period), what is the probability that it reaches location B? What is the expected path length and the minimal/expected time needed to cover that distance?

The computation of the mean and standard deviation of directions, $\bar{\theta }$ and $\sigma _\theta$, respectively, is as follows^44^: $\cos \bar{\theta }\equiv \bar{c}/\bar{r}$ or, equivalently, $\sin \bar{\theta }\equiv \bar{s}/\bar{r}$, and $\sigma _\theta ^2=-2\log \bar{r}$, where $\bar{c}\equiv \overline{\cos \theta }$, $\bar{s}\equiv \overline{\sin \theta }$, $\bar{r}^2=\bar{c}^2+\bar{s}^2$ and overbar denotes averaging over the whole sample. For $\theta$ distributed uniformly over the interval $[0,2\pi )$, $\sigma _\theta \approx 81^\circ$.

From the monthly-mean atmospheric circulation indices, available in the period 1950–present from the NOAA Climate Prediction Center https://www.cpc.ncep.noaa.gov/data/teledoc/telecontents.shtml, hte following ones are used in this analysis: North Atlantic Oscillation (NAO), East Atlantic (EA), East Atlantic/Western Russia (EAWR), Scandinavian (SCAND), and Arctic Oscillation (AO), see Supplementary Note S3. By construction, the circulation indices (apart from the AO) are uncorrelated, their mean equals zero and standard deviation equals one.

## Supplementary Information

Supplementary Information.

## Data Availability

All data used as input for this work are freely available through the Copernicus Marine Service (https://marine.copernicus.eu/) and the International Arctic Buoy Programme (IABP; https://iabp.apl.uw.edu/). The data created in this study, either in the form of a full dataset with particle trajectories (500 GB in volume) or a summary with path statistics, can be obtained from the corresponding author upon request.
