# Supplementary material for: Typical and anomalous pathways of surface-floating material in the Northern North Atlantic and Arctic Ocean
Source: Sci Rep. 2022 Nov 28;12:20521. doi: 10.1038/s41598-022-25008-5 (PMC9705288; doi:10.1038/s41598-022-25008-5)
Supplement: Supplementary file 1 — Supplementary Information. [file 41598_2022_25008_MOESM1_ESM.pdf]

Supplementary Material to

"Typical and anomalous pathways of surface-floating material in the northern North Atlantic and Arctic Ocean"

Agnieszka Herman, Jan Marcin Wesławski

Corresponding author:
Agnieszka Herman
Institute of Oceanology, Polish Academy of Sciences, Sopot, Poland
Email: agaherman@iopan.pl

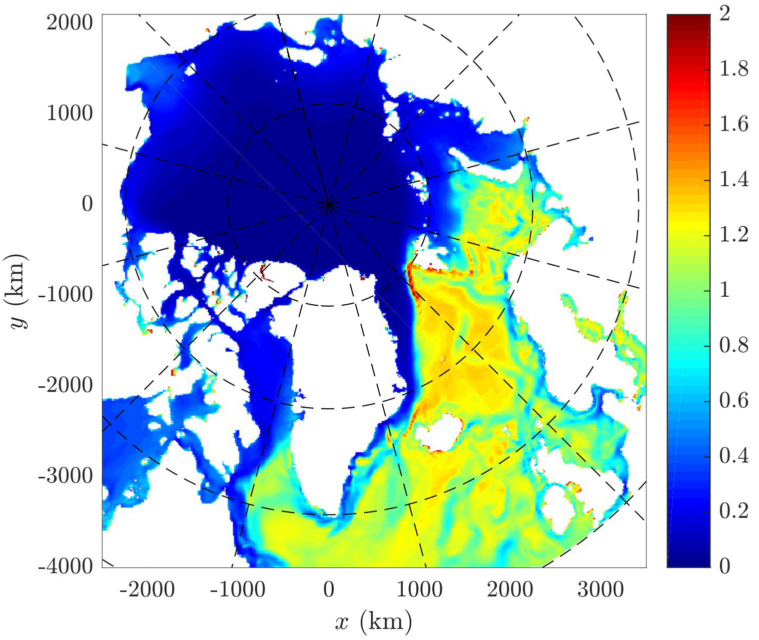

Supplementary Figure 1: The 1993–2020 mean ratio of the Stokes drift amplitude $|\mathbf{u}_S|$ to the amplitude of wind current $|\mathbf{u}_c|$ (see Fig.2c,f,i in the main text). Map created with Matlab version 2016b, `https://www.mathworks.com/`.

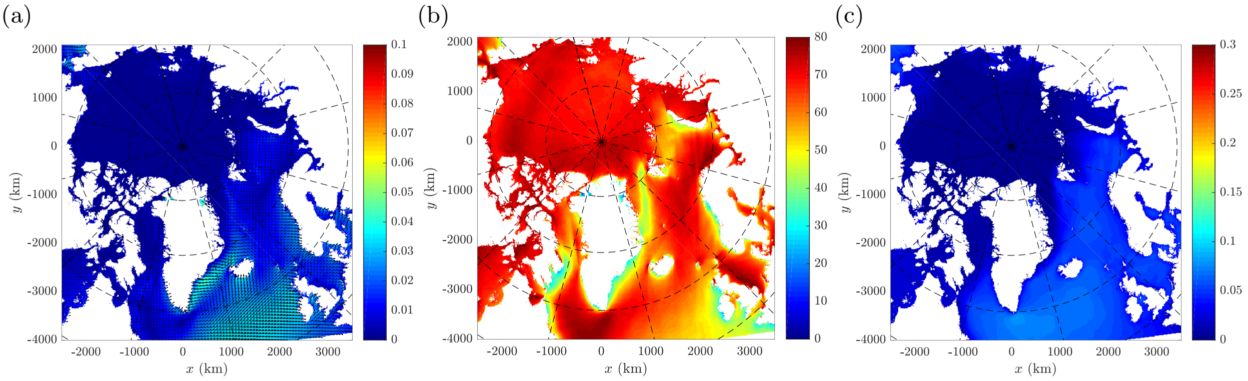

Supplementary Figure 2: The statistics of surface Stokes current velocities, computed from integral wave parameters (significant wave height, peak period and mean wave direction): vector mean velocity (a; in m$\cdot$s^{-1}), standard deviation of current direction (b; in degr) and mean amplitude (c; in m$\cdot$s^{-1}). For analogous maps of Stokes velocities computed from full wave energy spectra, see Fig.2d–f in the main text. (Maps created with Matlab version 2016b, `https://www.mathworks.com/`.)

Supplementary Note S1. The role of the diffusion coefficient K_h

As noted in the main text (see Methods), sensitivity simulations have been performed for trajectories originating at ICE in order to assess the influence of the value of the diffusion coefficient K_h on the modelled particle trajectories. Three values of K_h have been tested, $K_h = 29\ \mathrm{m}^2{\cdot}\mathrm{s}^{-1}$ (used in the simulations described in the main text), $K_h = 290\ \mathrm{m}^2{\cdot}\mathrm{s}^{-1}$ and $K_h = 2.9\ \mathrm{m}^2{\cdot}\mathrm{s}^{-1}$.

Supplementary Fig. 3 shows the corresponding probabilities of drifters reaching from ICE to the remaining 15 destination regions (see also connection matrix in Fig. 3a in the main text). As can be seen, for all connections with probabilities higher than 0.1%, their values obtained with the three different K_hs are very similar and remain at the level below 1%. Only connections to FJL, GLN and SBW show larger differences, but those destinations are reached by too few drifters for the statistics to be robust (this is the reason those connections are not analyzed in the discussion in the main text). Analogously, the statistics of the minimum and median drift times for connections from ICE remain largely unaffected Supplementary Fig. 4), with the same exceptions as those described above – although, as expected, overall larger K_h lead to slightly shorter times, and the differences in times obtained with different K_h get more pronounced for longer trajectories, e.g., those to BEI or SVA.

The time series of release and landing events, shown for two examples (NOR and SVA) in Supplementary Fig. 5, exhibit some differences for different K_h – again, as expected, the peaks in those time series are generally smaller for smaller K_h and *vice versa* – but, crucially, the differences are very small between $K_h = 29\ \mathrm{m}^2{\cdot}\mathrm{s}^{-1}$ and $K_h = 290\ \mathrm{m}^2{\cdot}\mathrm{s}^{-1}$, the two values that can be seen as limits of K_h reported in the literature.

Overall, the results of the sensitivity tests show rather limited influence of K_h on the statistical properties of the modelled trajectories, suggesting that the results obtained with $K_h = 29\ \mathrm{m}^2{\cdot}\mathrm{s}^{-1}$, discussed in the main body of this paper, can be treated as representative for a wide range of realistic values of the diffusion coefficient.

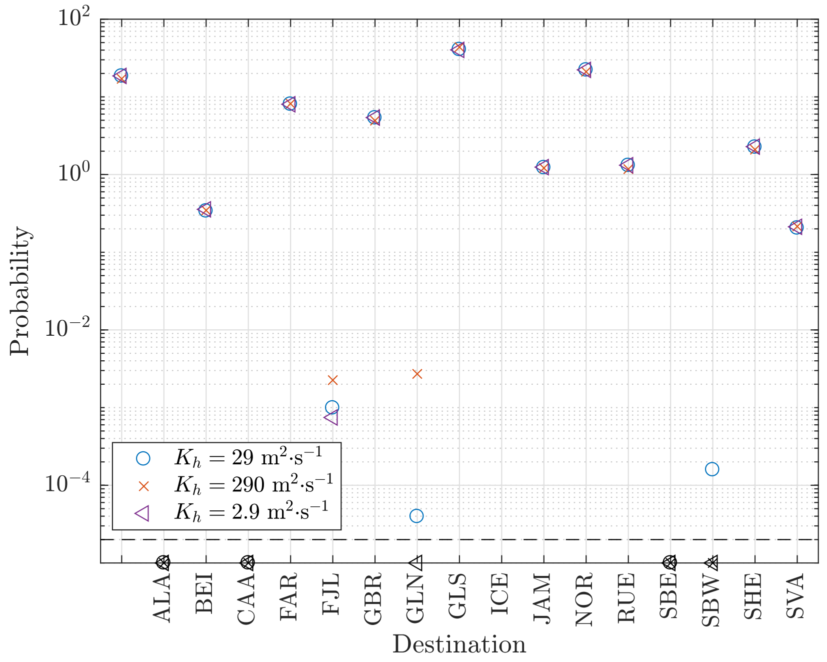

Supplementary Figure 3: The influence of K_h on the fraction (in percent) of particles from ICE reaching the 15 destination regions considered. The left-most, unlabelled value corresponds to particles that left the domain of study or are still adrift after the maximum time of 3 years.

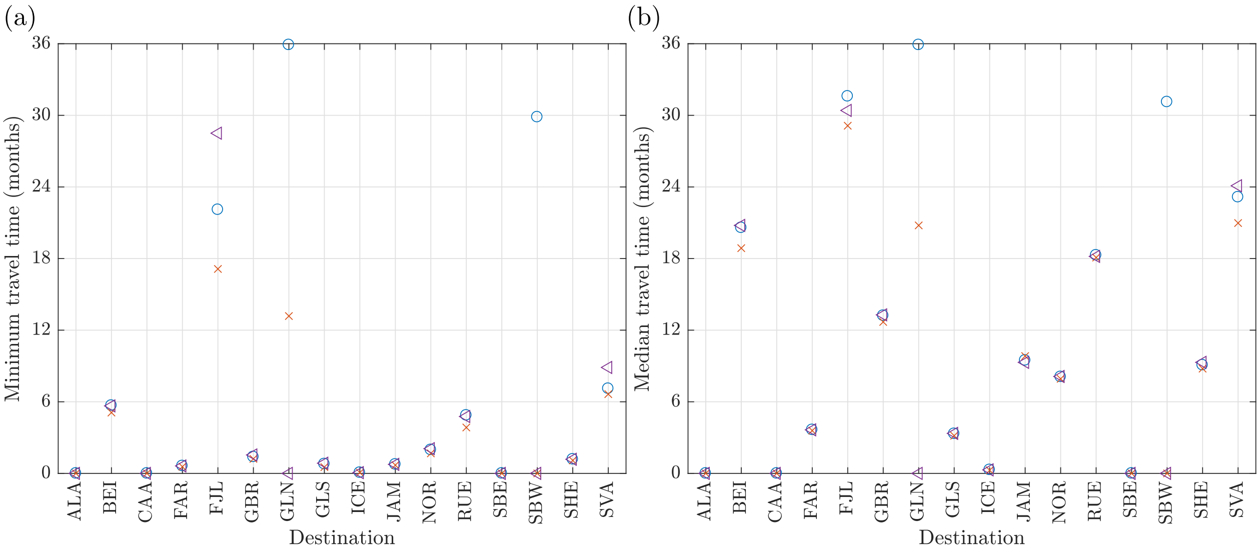

Supplementary Figure 4: The minimum (a) and median (b) drift time in months between ICE and the other 15 regions considered. Meaning of symbols as in Supplementary Fig. 2.

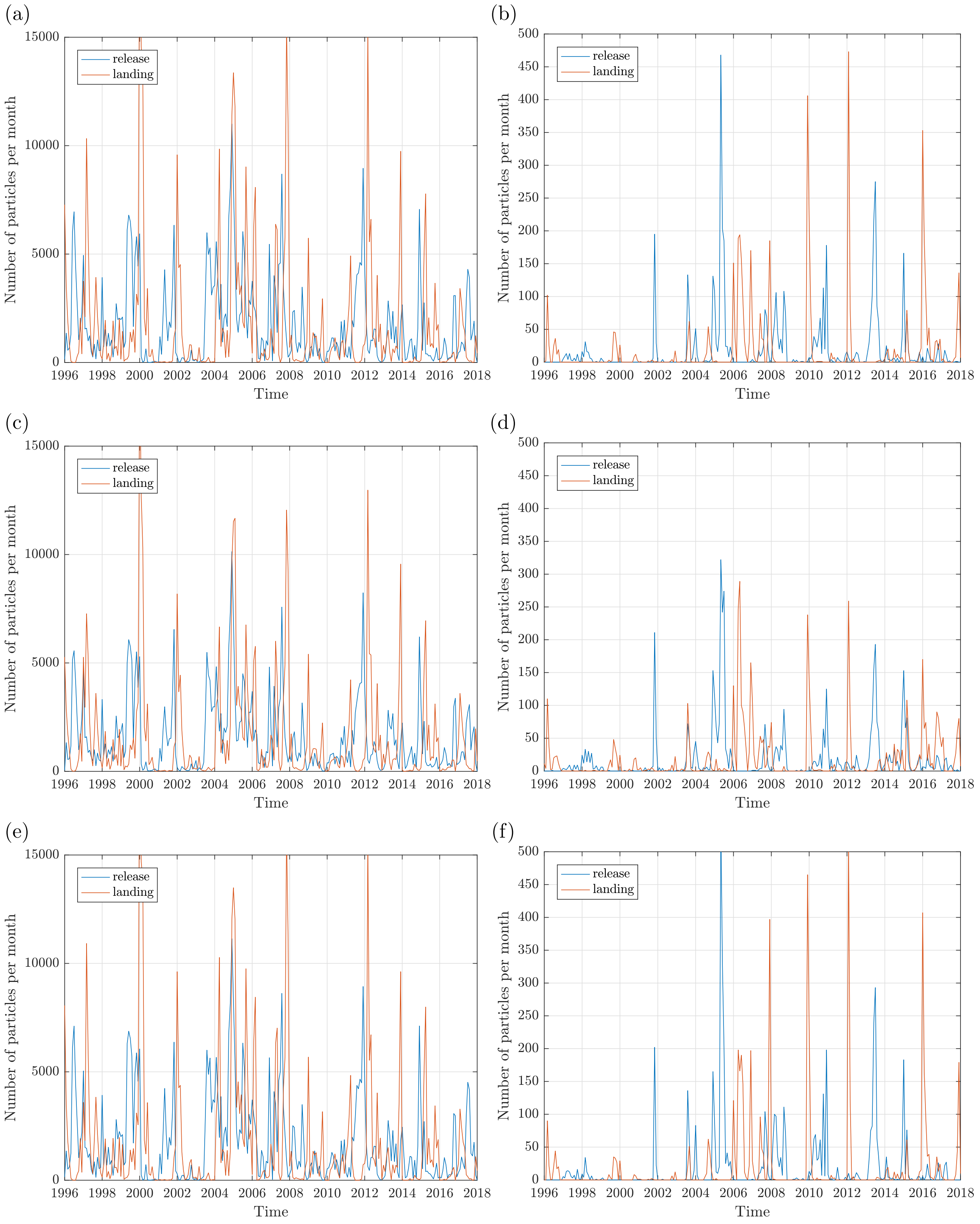

Supplementary Figure 5: Time series of monthly release (blue) and landing (red) events for particles originating at ICE and reaching NOR (a,c,e) and SVA (b,d,f). Results obtained with $K_h = 29$ m$^2\cdot$s^{-1} (a,b), $K_h = 290$ m$^2\cdot$s^{-1} (c,d) and $K_h = 2.9$ m$^2\cdot$s^{-1} (e,f). Note different ranges of the y-axis in (a,c,e) and (b,d,f).

Supplementary Note S2. Validation against buoy data

As mentioned in the main text, buoy tracks from the International Arctic Buoy Programme (`https://iabp.apl.uw.edu/`) are used here as a source of *in situ* data for validation of the model. The usage of IABP data has several limitations due to the fact that the buoys' drift speeds and directions are a net result of forces from the uppermost ocean and the lowermost atmosphere – they do not strictly fulfill our assumptions of drifters subject to forcing at depth $z = 0$. Moreover, the influence of the wind and current forcing on the buoys tracks is very hard to estimate, as several types of buoys are used, with different mass, shape, and oceanographic/atmospheric instruments attached to them. Also, the role of winds and currents certainly changes with changing ice conditions (ice type, concentration, etc.). Nevertheless, the IABP buoy tracks are routinely used in analyses of sea ice drift patterns, dispersion etc., not only in the central Arctic, but also in very dynamic regions as e.g. the Fram Strait/East Greenland Current. They are also used for validating satellite products of ice motion (including the data assimilated in HYCOM). Therefore, keeping the above limitations in mind, we are convinced that the comparison of our trajectories with the IABP data provides useful insights into the quality and reliability of our results.

In the following analysis we use LEVEL 2 data (i.e., after basic quality control), available in the years 2015–2020, supplemented with LEVEL 1 data from 2014 in order to increase the number of data points (and, in particular, the number of tracks in the open-water North Atlantic part of the domain of study). The time series of buoys' positions are interpolated in time onto the 12:00 hour of each day, and those daily time series are used for model validation. After removing tracks with unrealistically high displacements and/or obviously incorrect positions, a total of 387 tracks are retained for further analysis, ranging from 1 to 39 months in duration (with a median value of 209 days) and from 1 to 15000 km in length (with a median value of 1960 km). The initial and final positions of those tracks are shown in Supplementary Fig. 6. For each track, the model is run 20 times (10 times without the Stokes drift, and 10 times with Stokes drift taken into account), initialized from a location randomly selected from within a square region with side length equal to 5 km, centered at the initial buoy location (i.e., according to the same procedure as used in the main simulations analyzed in the text of the paper).

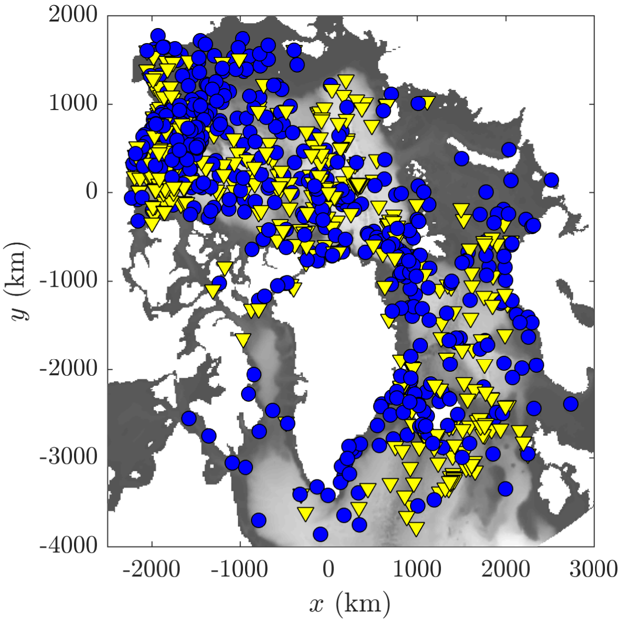

Supplementary Figure 6: Positions of the initial (yellow triangles) and final (blue circles) positions of buoys used in the analysis described in Supplementary Note S2. (Map created with Matlab version 2016b, `https://www.mathworks.com/`.)

As the main measure of the model performance, the relative error d_{best}/L has been selected, where d_{best} denotes the distance (in meters) between the simulated and observed final positions of the buoy, computed for the 'best' path, and L denotes the total length (in meters) of the observed buoy path. The ratios d_{best}/L are computed separately for simulations with and without Stokes drift. The summary of the d_{best}/L statistics is shown in Supplementary Fig. 7 in the form of the respective pdfs and cdfs. As can be seen, in $\sim$50% of the cases the simulated final positions have errors smaller than 10%, and for close to 80% of cases the error is smaller than 20%. In general, the pdfs of d_{best}/L can be well described by exponential curves. Notably, when all trajectories are taken into account, the role of the Stokes drift vanishes – the exponential fits for the results obtained with/without the Stokes drift are essentially the same. The reason for that is the fact that the majority of IABP buoys are located in sea ice, when the role of waves, and thus also the role of the associated Stokes drift, is negligible. When analogous pdfs/cdfs are computed for open-water cases (i.e., trajectories located in the North Atlantic or in the ice-free regions of the Arctic Ocean during the summer), the model performance is significantly better when the Stokes drift is taken into account (compare the blue and red curves in Supplementary Fig. 7c.d).

Importantly, as well, the bias of the length of simulated trajectories for simulations with Stokes drift is very small: the mean normalized difference between the total modelled and observed path length, $(L_{\mathrm{s}}-L)/L$, equals -0.07, and the corresponding standard deviation 0.56. The bias for model version without Stokes drift is much higher and equals -0.26, in accordance with the fact that the paths without the contribution of wave-induced drift are usually shorter. The scatter in that case is similar and equals 0.53.

In order to get a better insight into the model performance and behaviour, we show selected examples of trajectories in Supplementary Figs 8–14. In all figures, the initial position and the trajectory of the IABP buoy are shown with a yellow triangle and a red curve, respectively; the simulated trajectories and their final positions are marked with magenta curves and white circles (version without Stokes drift) and with blue curves and blue circles (version with Stokes drift), respectively. The observations made based on those examples (and several other tracks, which are not shown) are as follows:

- In most parts of the central Arctic Ocean, the model accurately reproduces both drift speed and direction of the buoys (Supplementary Fig. 8), presumably thanks to the assimilation of satellite sea ice drift data used in the ocean circulation model, on which our simulations are based.

- In the Chukchi and East Siberian Seas, and in particular in the region around the Wrangel Island, the trajectories often have very irregular shapes, with several loops and rapid shifts of direction, combined with a relatively low average drift speeds. Although the modelled trajectories generally reproduce those overall path characteristics, there is a large scatter between individual paths, especially during periods of weak atmospheric/oceanic forcing and low sea ice concentration. Nevertheless, in most cases the mean drift direction is correctly reproduced (Supplementary Fig. 9).

- The simulated trajectories along the east coast of Greenland usually reveal large scatter in terms of their landing locations, as the 'fate' of individual paths is very sensitive to their distance from shore and small perturbations in forcing. However, in most cases (including the examples in Supplementary Fig. 10), some of those trajectories reach land very close to the observed landing location. In this case, as in the one described in the previous point, the drift speeds computed without Stokes drift generally tend to be too slow compared to observations.

- The model captures also drift paths within the cyclonic recirculation region to the south of the Denmark Strait (Supplementary Fig. 11).

- Reproducing trajectories during weak and/or highly variable wind and wave forcing is a challenge (Supplementary Fig. 12 shows an example from winter-time in the Barents Sea). However, by definition, those situations contribute very little to the long-distance transport between coastal regions, which is the main subject of the paper.

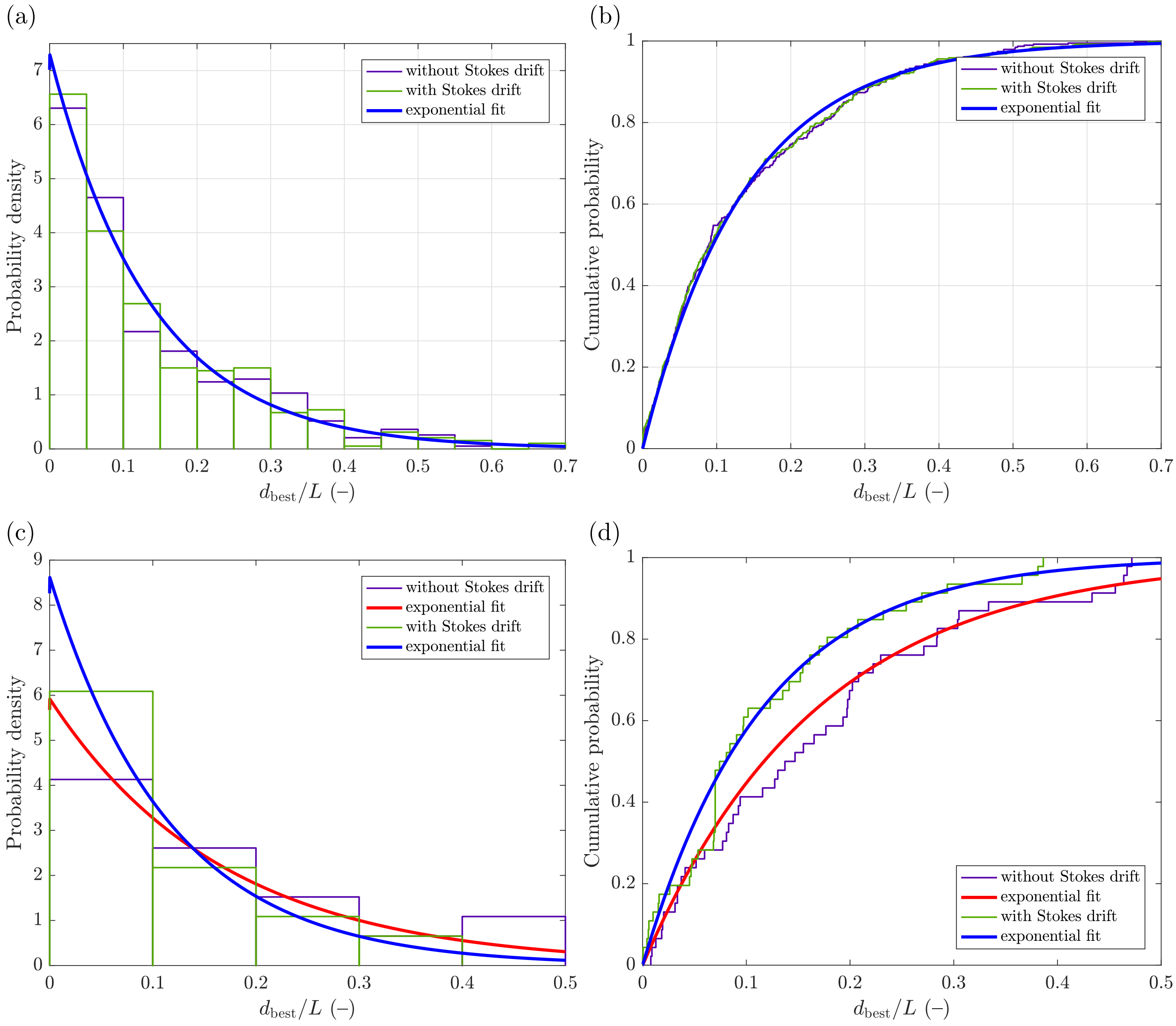

Supplementary Figure 7: A summary of the model validation against IABP data for all buoy tracks (a,b) and for buoy tracks in ice-free areas (c,d): probability density functions (a,c) and cumulative probability distributions (b,d) of the relative model error, defined as the ratio between d_{best} (defined as the minimum distance, among all simulated paths, between the final predicted and observed buoy position) and the total path length L. Note that both versions of the exponential fit in (a,b) are essentially identical, so that only one fit is shown. Bin width and number of data points in (a,b) equal 0.05 and 387, respectively; in (c,d), the values are 0.1 and 56. See text for more details.

- In regions without sea ice and/or with low ice concentration, the Stokes drift enhances the average drift speeds (Supplementary Fig. 13a,b) and contributes to the landing of particles, as in the example in Supplementary Fig. 13c, where none of the no-Stokes trajectories, but nine out of ten of the Stokes-trajectories reached land (some in vicinity of the observed landing location).
- Finally, there are several cases when the model failed to reproduce the observed trajectories, as in the examples in Supplementary Fig. 14. Those very large discrepancies are typically associated with model inaccuracies in regions located at the boundaries of two neighboring circulation cells, like e.g., the Beaufort Gyre and the Transpolar Drift leading to the Fram Strait (Supplementary Fig. 14b). As the statistics presented earlier in this section shows, those cases constitute only a small amount of the total number of trajectories.

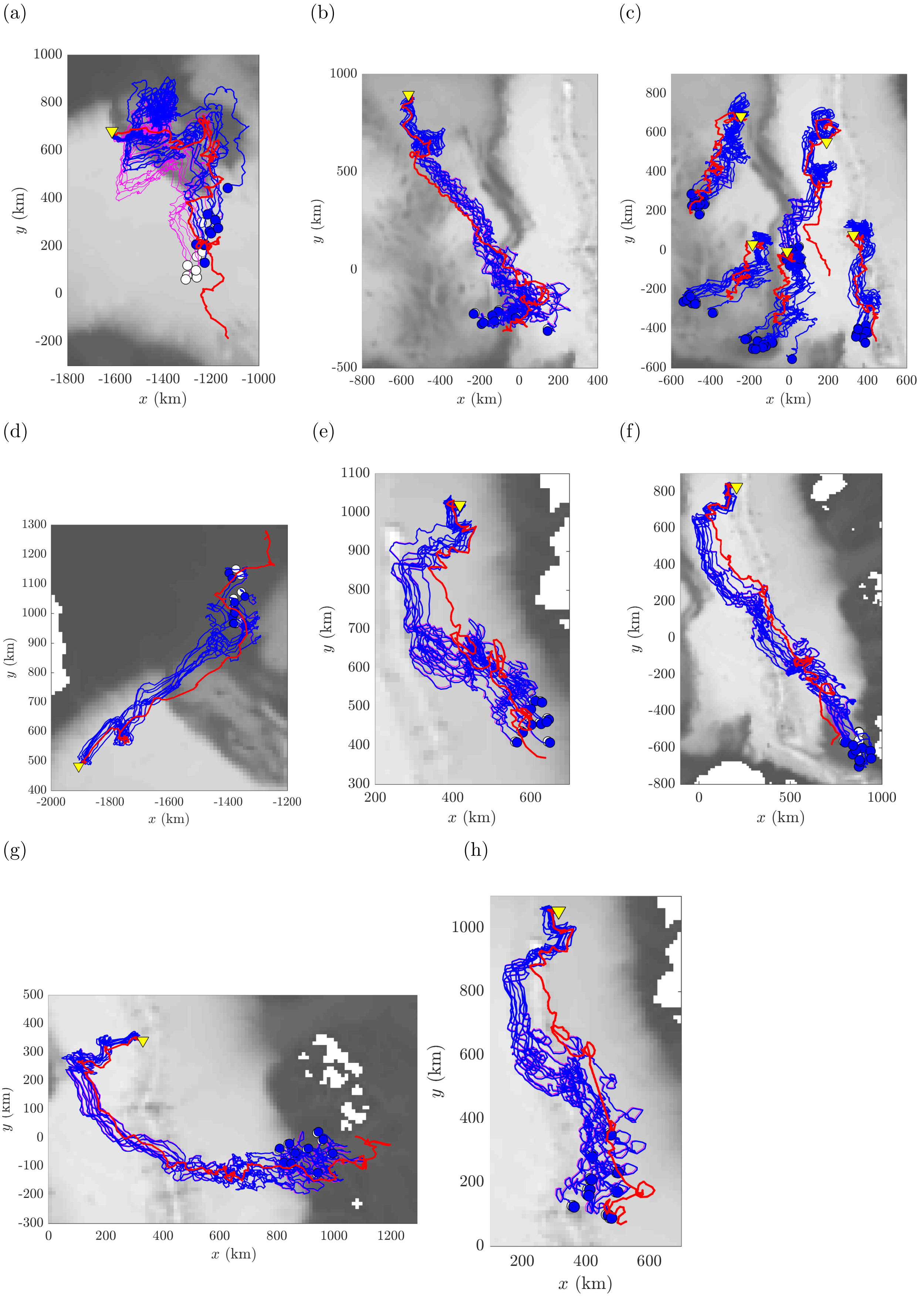

Supplementary Figure 8: The initial positions (yellow triangles) and trajectories (red curves) of selected IABP buoys in the Arctic Ocean, and the corresponding simulated trajectories and their final positions (magenta curves and white circles: version without Stokes drift; blue curves and blue circles: version with Stokes drift): 21.09.2016–15.08.2017 (a), 13.09.2018–11.01.2020 (b), a group of buoys active over 09.2015–03.2016 (c), 01.04.2014–08.09.2014 (d), 10.01.2014–08.09.2014 (e), 10.01.2014–31.12.2014 (f), 10.01.2014–26.102014 (g), 10.01.2014–09.10.2014 (h). (Maps created with Matlab version 2016b, `https://www.mathworks.com/`.)

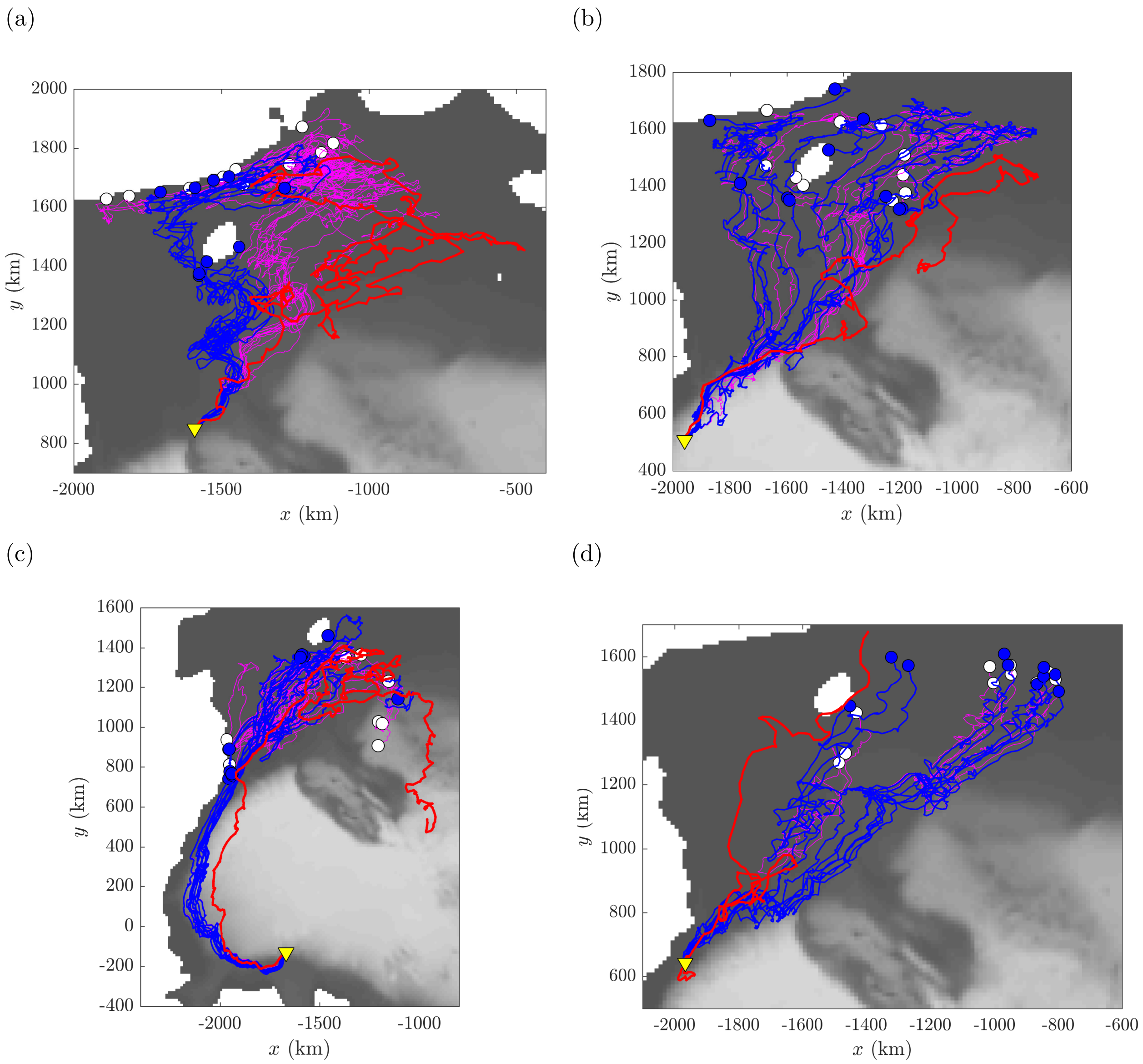

Supplementary Figure 9: As in Supplementary Fig. 8, for trajectories in the region of Beaufort/Chukchi/East Siberian Sea: 18.07.2015–15.08.2017 (a), 15.07.2015–25.09.2016 (b), 30.09.2015–01.10.2017 (c), 09.03.2014–07.12.2014 (d). (Maps created with Matlab version 2016b, `https://www.mathworks.com/`.)

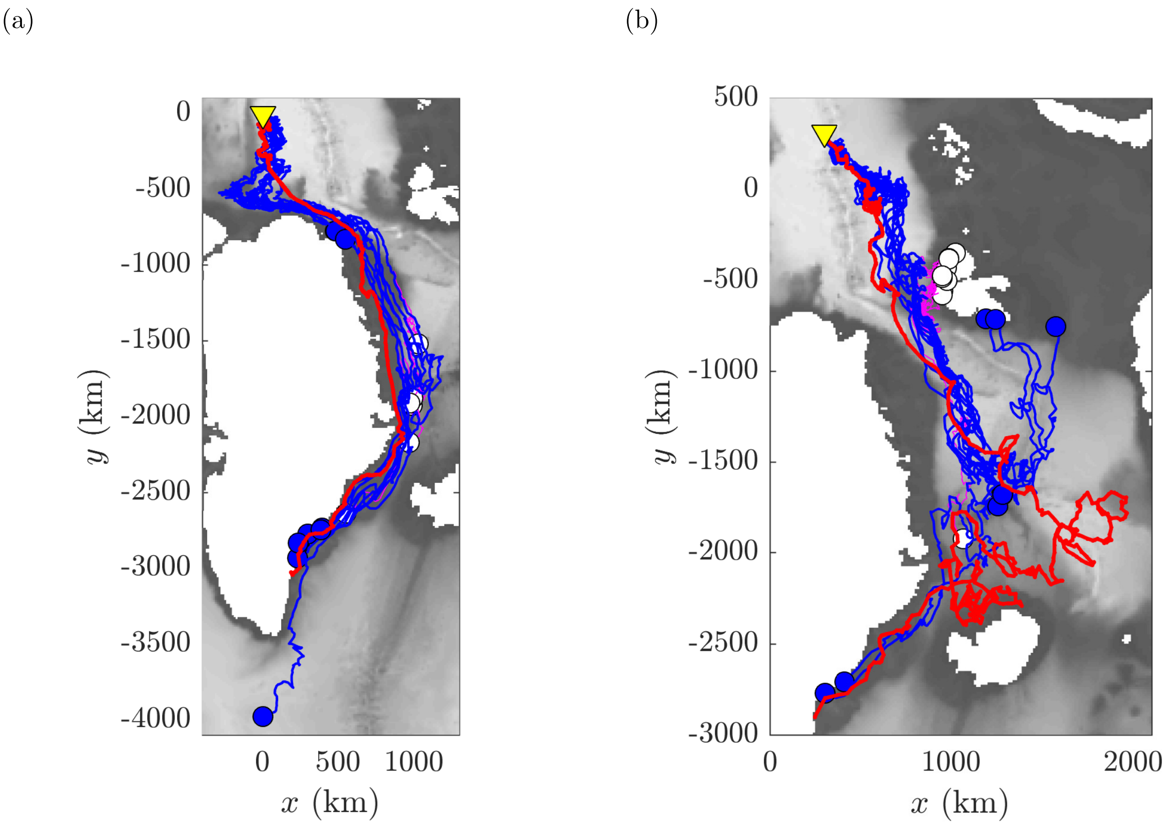

Supplementary Figure 10: As in Supplementary Fig. 8, for trajectories through the Fram Strait and along the east coast of Greenland: 20.08.2015–20.06.2017 (a), 19.04.2015–25.04.2017 (b). (Maps created with Matlab version 2016b, `https://www.mathworks.com/`.)

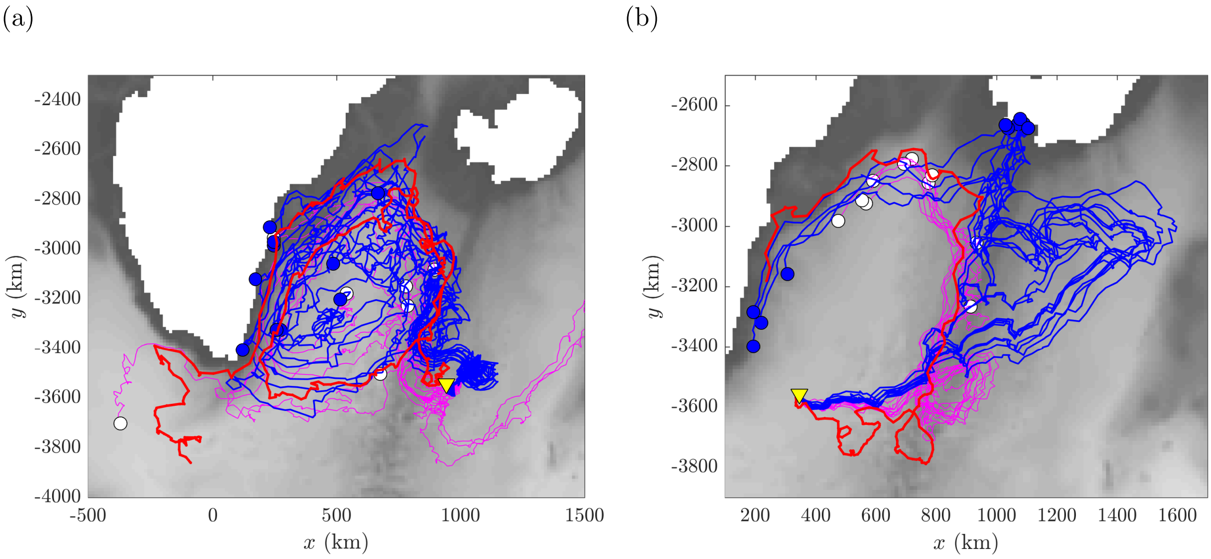

Supplementary Figure 11: As in Supplementary Fig. 8, for trajectories within the recirculation region to the southeast of Greenland: 26.03.2016–04.04.2017 (a), 11.08.2018–19.03.2019 (b). (Maps created with Matlab version 2016b, `https://www.mathworks.com/`.)

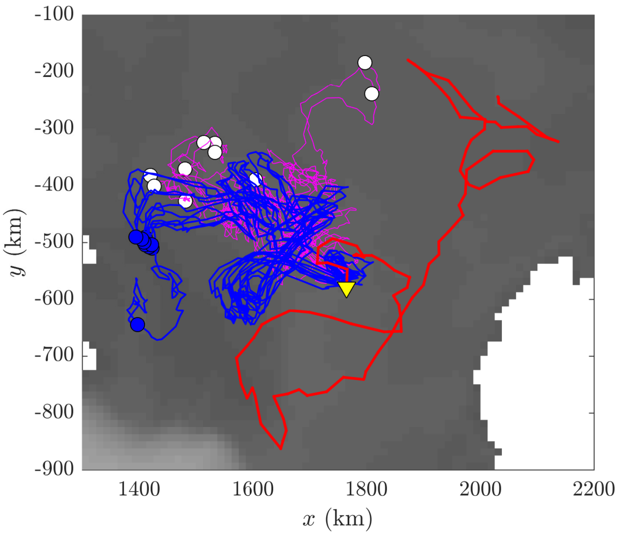

Supplementary Figure 12: As in Supplementary Fig. 8, for a trajectory in the Barents Sea 02.10.2014–03.04.2015. (Maps created with Matlab version 2016b, `https://www.mathworks.com/`.)

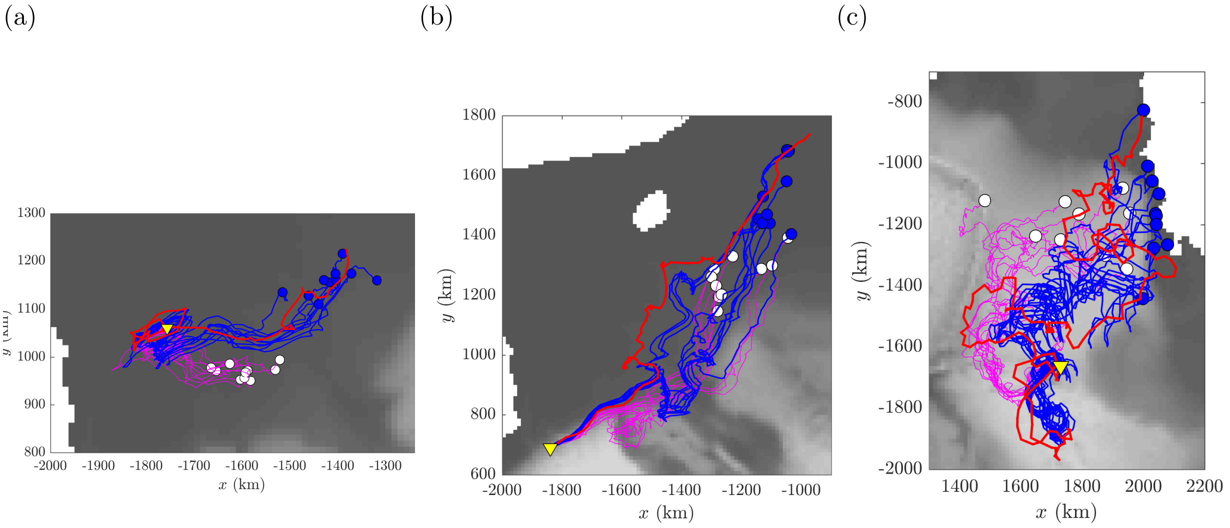

Supplementary Figure 13: As in Supplementary Fig. 8, for example trajectories in the Chukchi/East Siberian Sea (a,b) and northeast North Atlantic (c), with a significant role of the Stokes drift: 13.08.2018–25.10.2018 (a), 03.09.2017–20.12.2017 (b), 13.09.2018–14.04.2019 (c). (Maps created with Matlab version 2016b, `https://www.mathworks.com/`.)

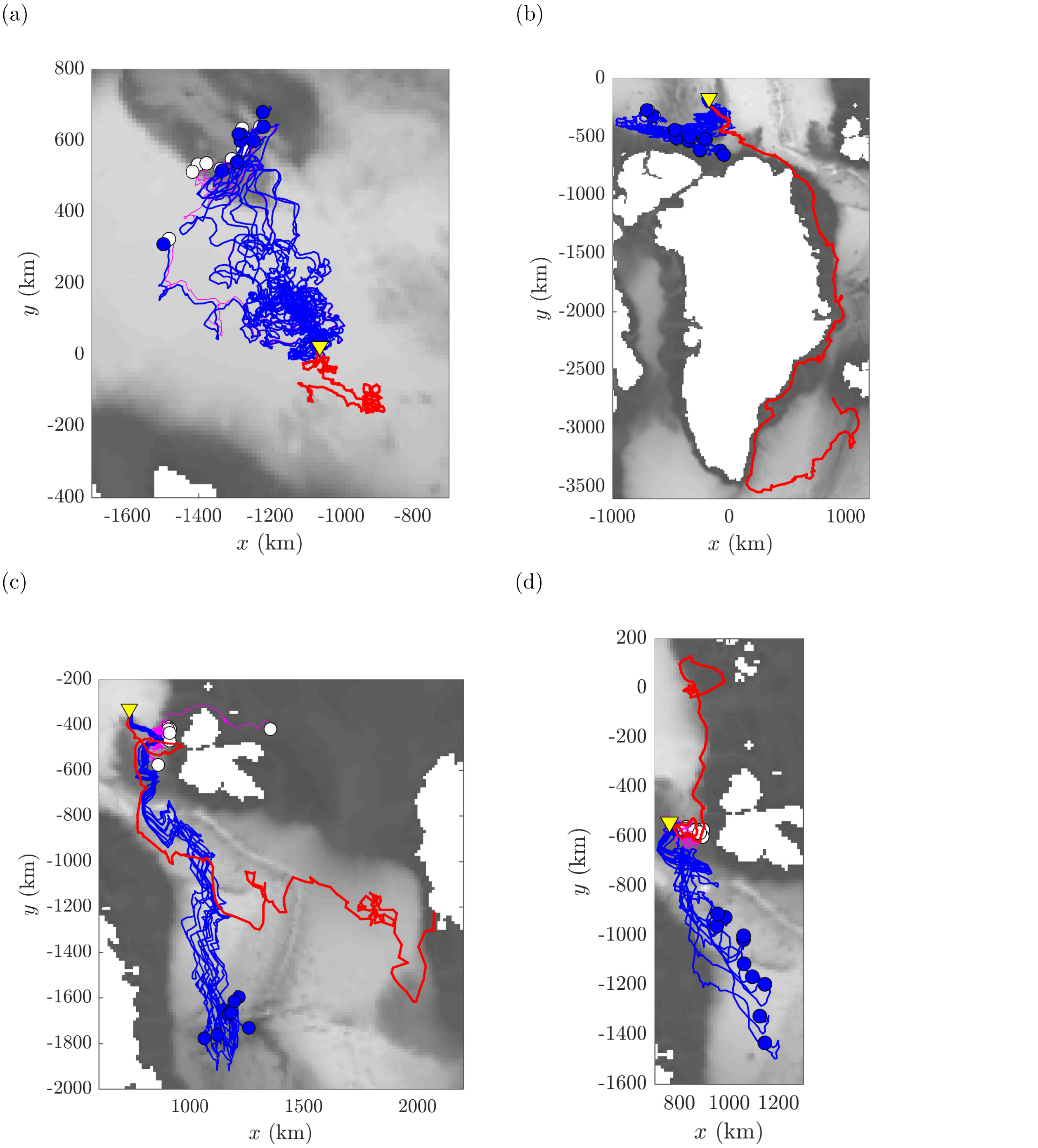

Supplementary Figure 14: As in Supplementary Fig. 8, for example trajectories that the model failed to reproduce: 04.10.2018–08.11.2019 (a), 14.04.2015–15.12.2016 (b), 19.09.2018–24.04.2019 (c), 10.08.2018–21.12.2018 (d). (Maps created with Matlab version 2016b, `https://www.mathworks.com/`.)

Supplementary Note S3. Atmospheric circulation indices

Supplementary Figs. 15 and 16 show the spatial patterns and the corresponding time series, respectively, of the four large-scale atmospheric circulation indices used in the main text to construct predictors of GBR→FAR and GBR→ICE transport events. Analogous plots for the Arctic Oscillation (AO) pattern and time series, discussed in the text, are shown in Supplementary Fig. 17.

Supplementary Figs. 18 and 19 show connection matrices, analogous to those in Fig. 3 in the main paper, with colours coding corresponding to linear correlation coefficients between the time series of the atmospheric circulation indices considered (a–e) and the monthly number of particles released and landed, respectively.

(a) NAO

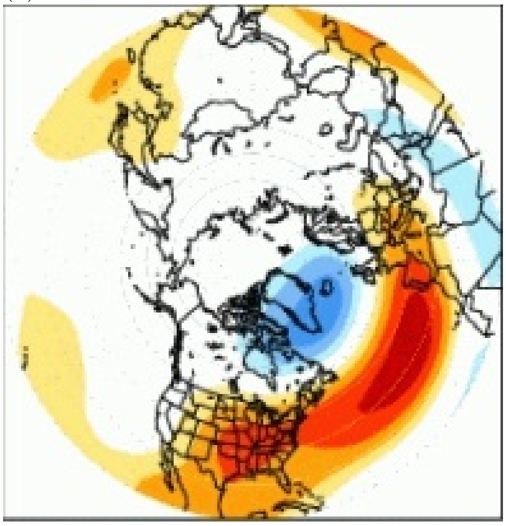

(b) EA

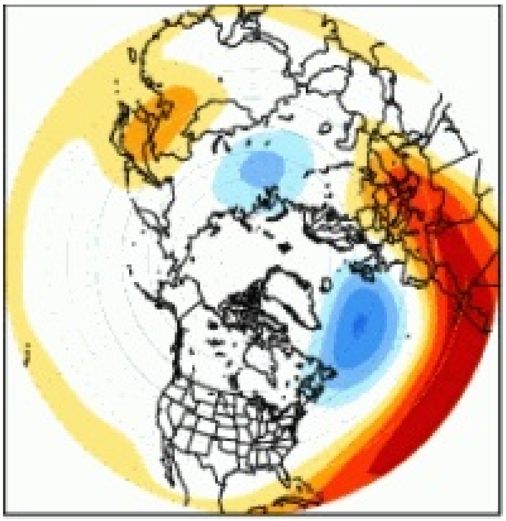

(c) EA/WR

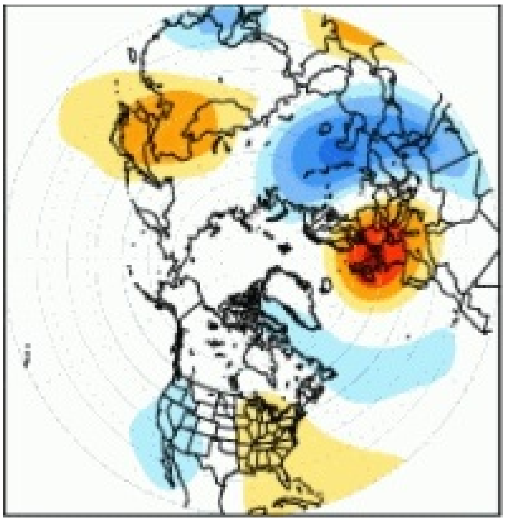

(d) SCAND

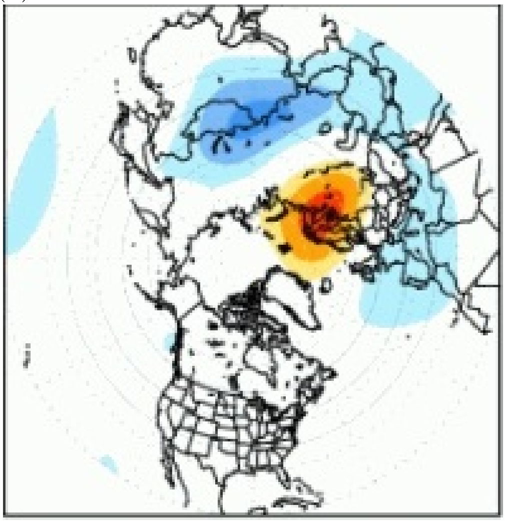

Supplementary Figure 15: Spatial patterns (for the month of January) associated with the four atmospheric circulation indices active over the North Atlantic and the European Arctic. Positive/negative values are shown in red/blue. Source: NOAA Climate Prediction Center, `https://www.cpc.ncep.noaa.gov/data/teledoc/telecontents.shtml`.

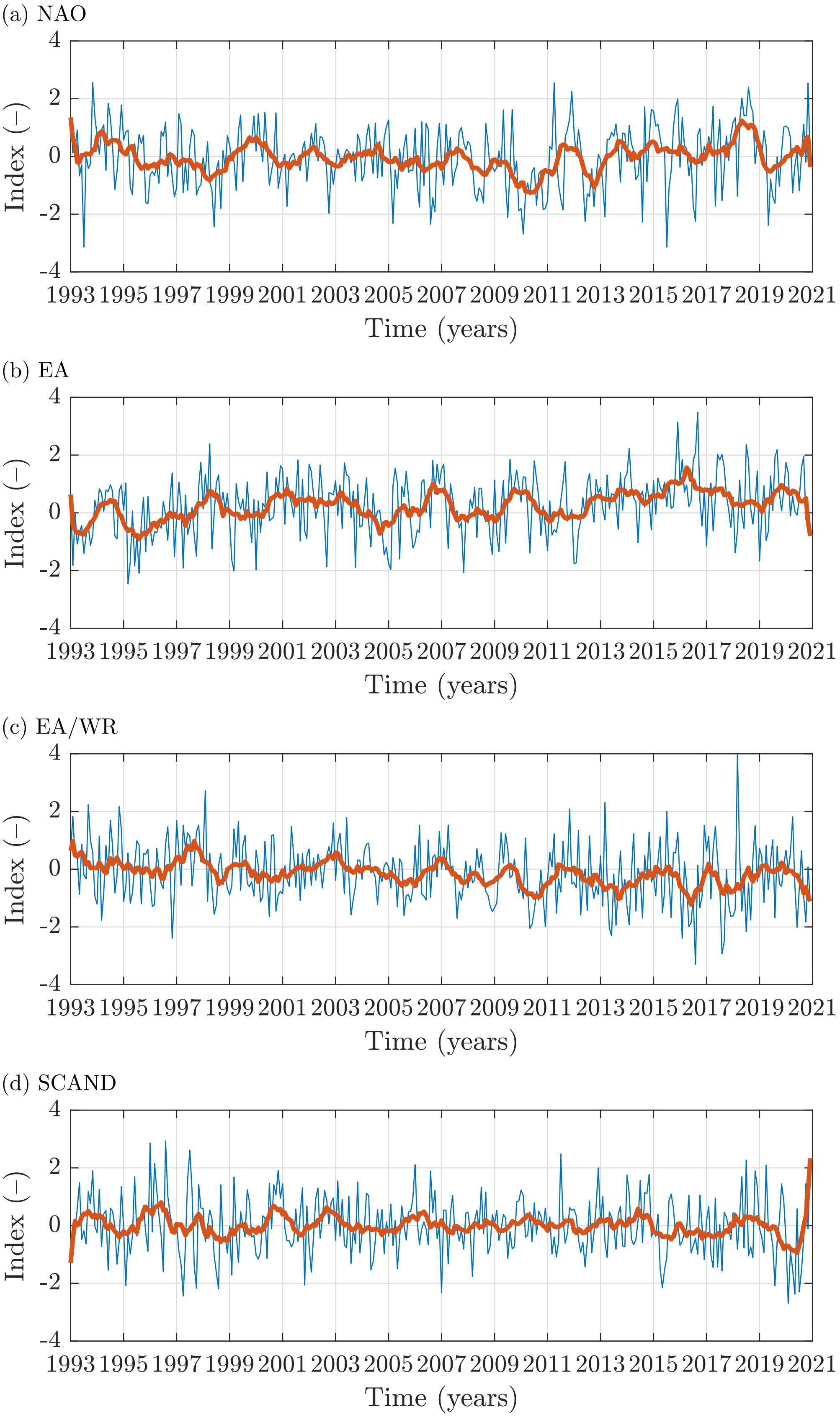

Supplementary Figure 16: Time series of monthly mean (blue) and 12-month smoothed (red) atmospheric circulation indices: NAO (a), EA (b), EA/WR (c) and SCAND (d). Data source: NOAA Climate Prediction Center, `https://www.cpc.ncep.noaa.gov/data/teledoc/telecontents.shtml`.

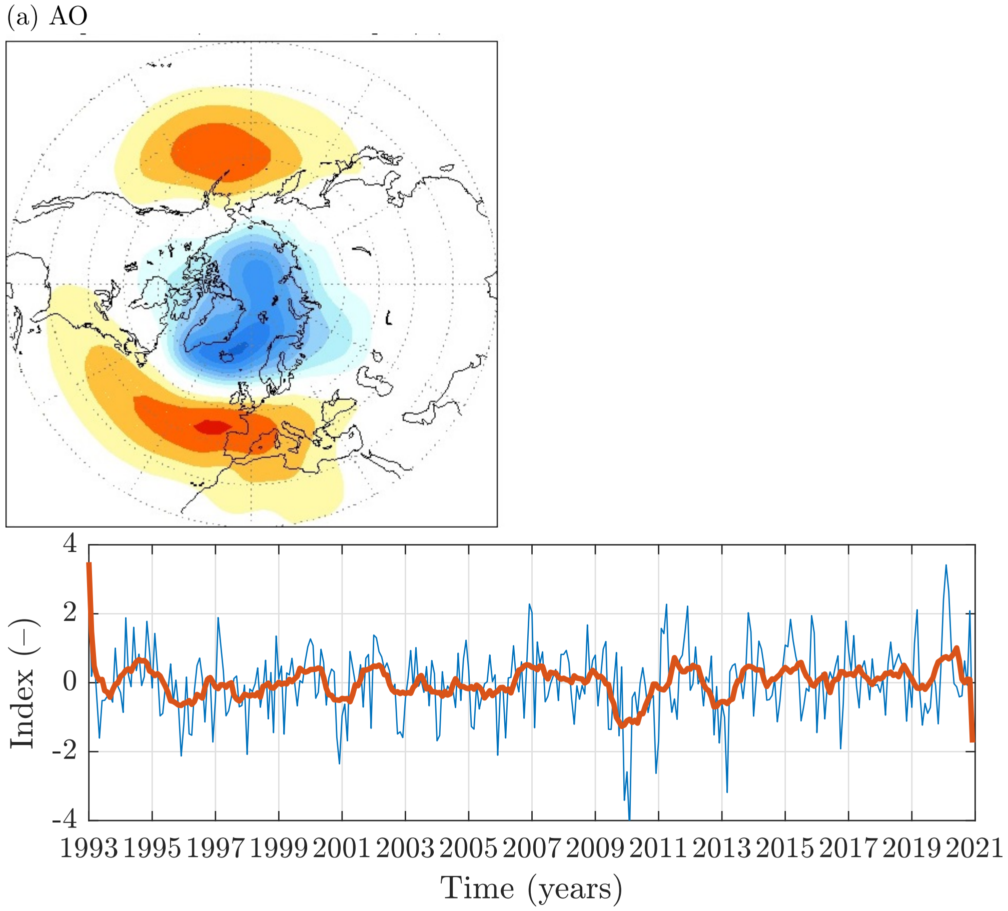

Supplementary Figure 17: Spatial pattern (a) associated with the Arctic Oscillation (AO) index and the time series of monthly mean (blue) and 12-month smoothed (red) AO (b). In (a), positive/negative values are shown in red/blue. Source: NOAA Climate Prediction Center, `https://www.cpc.ncep.noaa.gov/products/precip/CWlink/daily_ao_index/ao.shtml`.

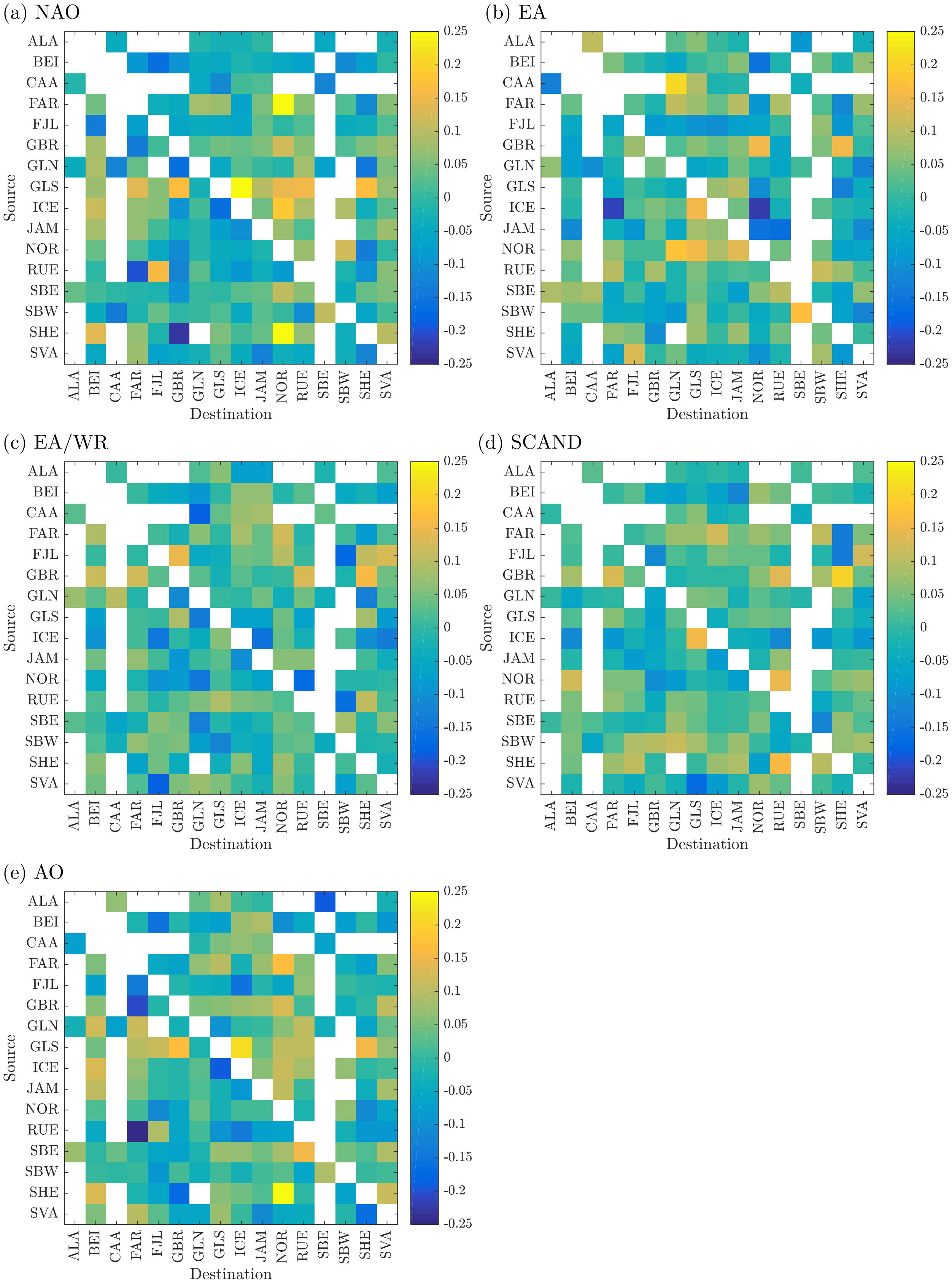

Supplementary Figure 18: Correlation coefficients (for all source–destination pairs) between monthly number of particles released from a given source and the five atmospheric circulation indices analyzed.

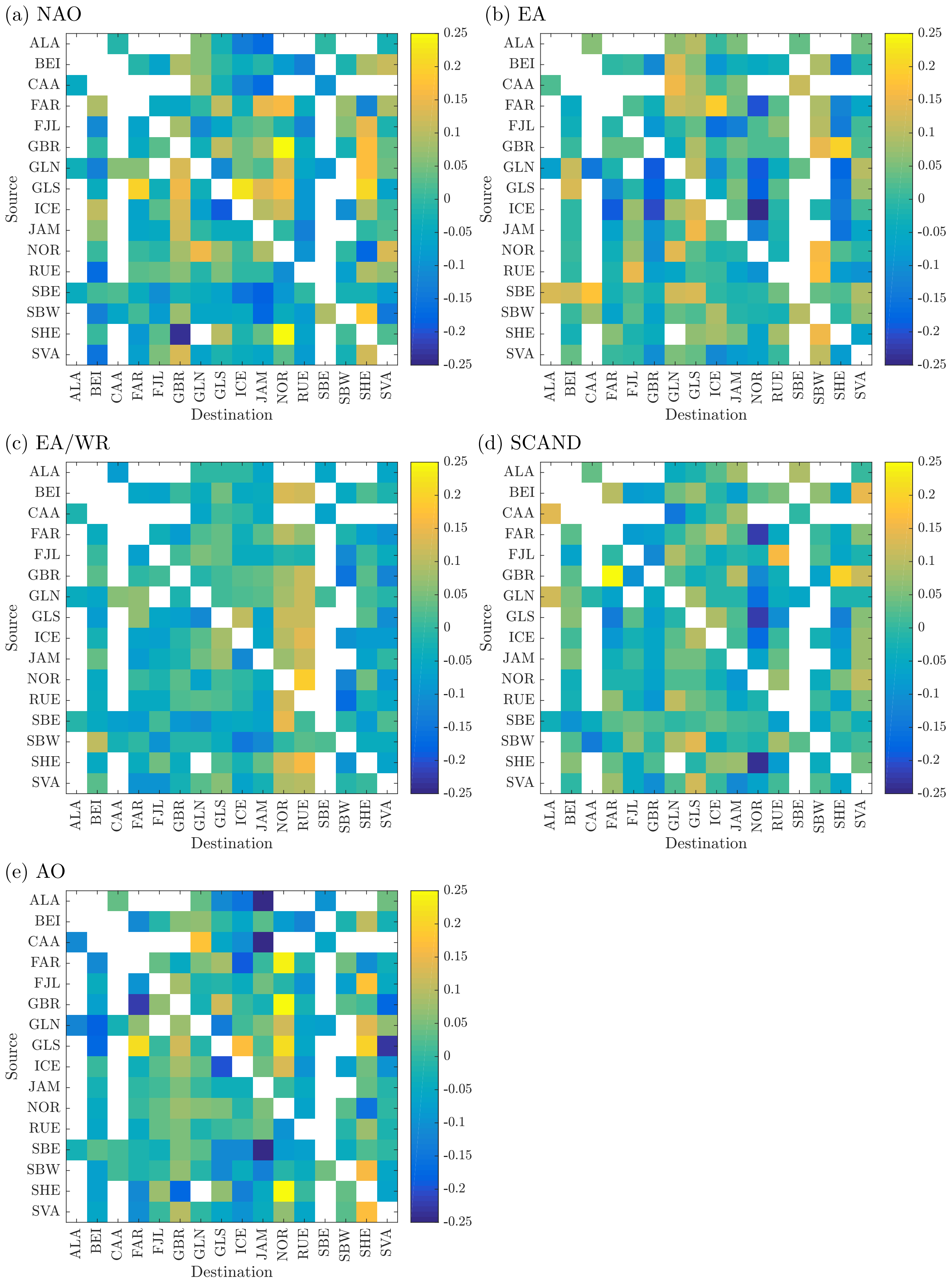

Supplementary Figure 19: As in Fig. 18, but for monthly landing times.

Supplementary Note S4. Spatial distribution of drift material

Supplementary Figs. 20–22 show maps of final positions of all particles released from the 16 regions considered (see main text for a discussion).

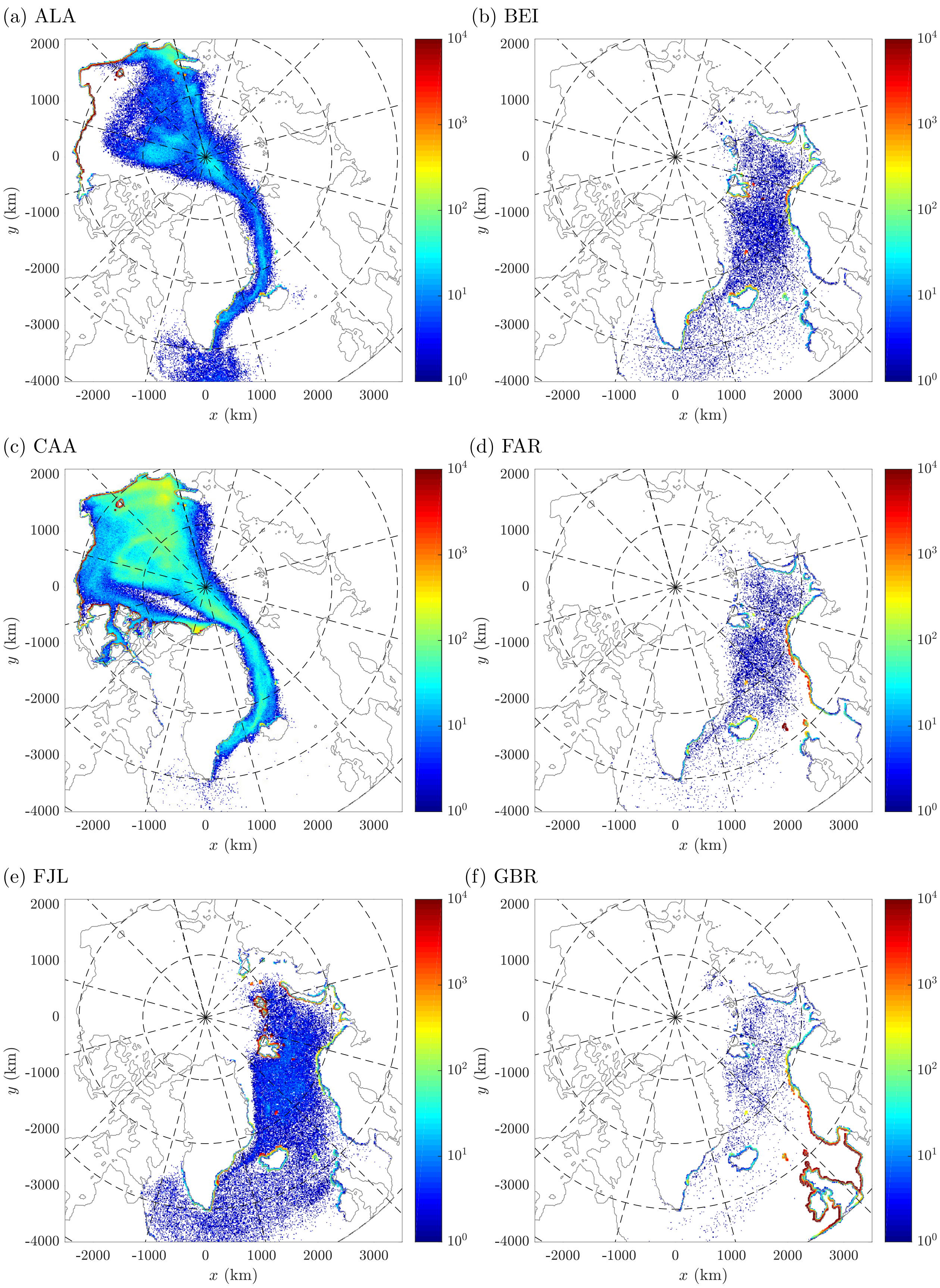

Supplementary Figure 20: Maps of the spatial density of final positions of all particles released from ALA, BEI, CAA, FAR, FJL, GBR (a–f) in the period 1993–2017 (in number of particles per grid cell, i.e., ~156 km^2). The color scale is logarithmic, grid cells with no particles are white. (Maps created with Matlab version 2016b, `https://www.mathworks.com/`.)

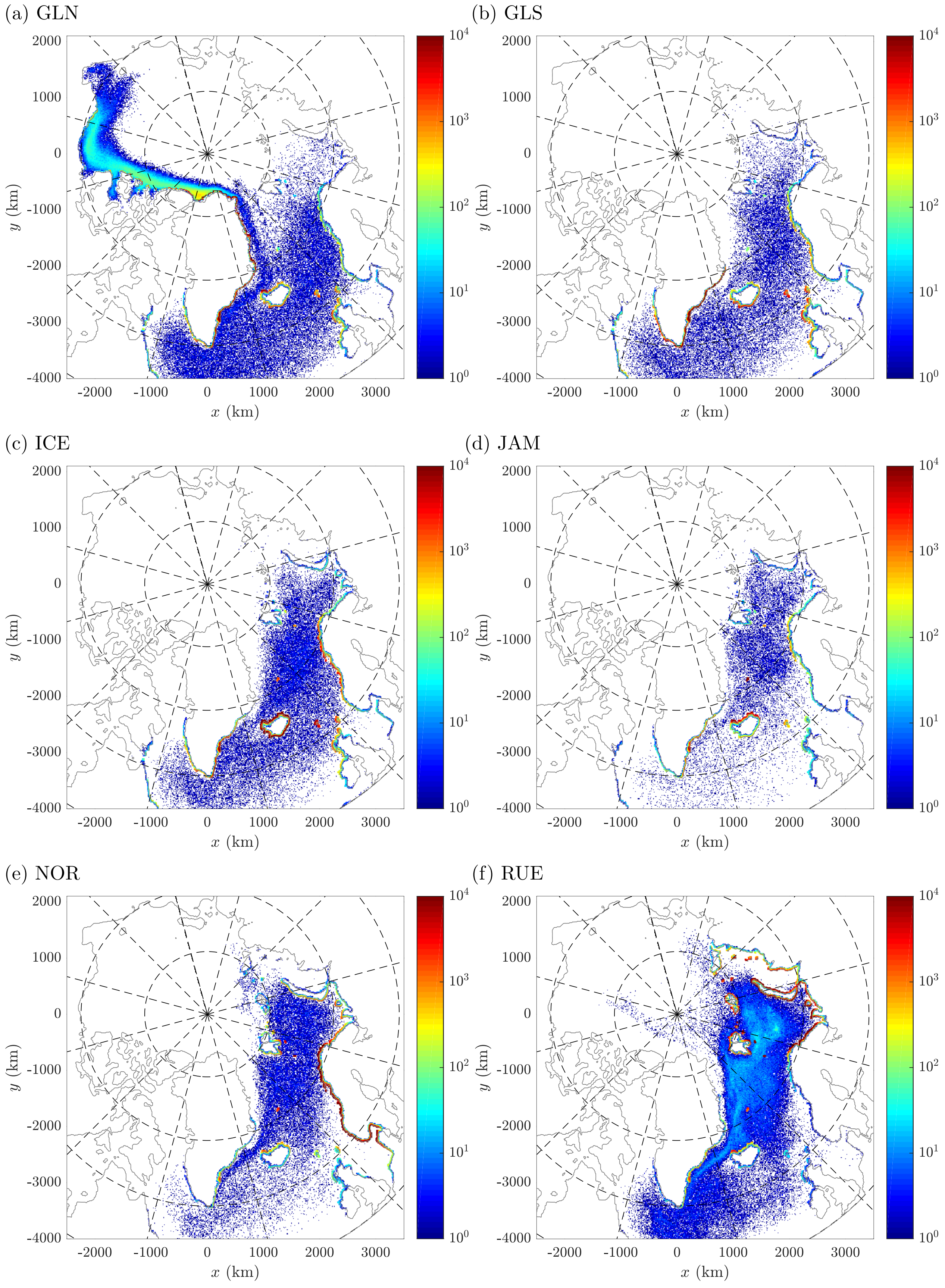

Supplementary Figure 21: As in Supplementary Fig. 9, but for particles released from GLN, GLS, ICE, JAM, NOR and RUE (a–f). (Maps created with Matlab version 2016b, `https://www.mathworks.com/`.)

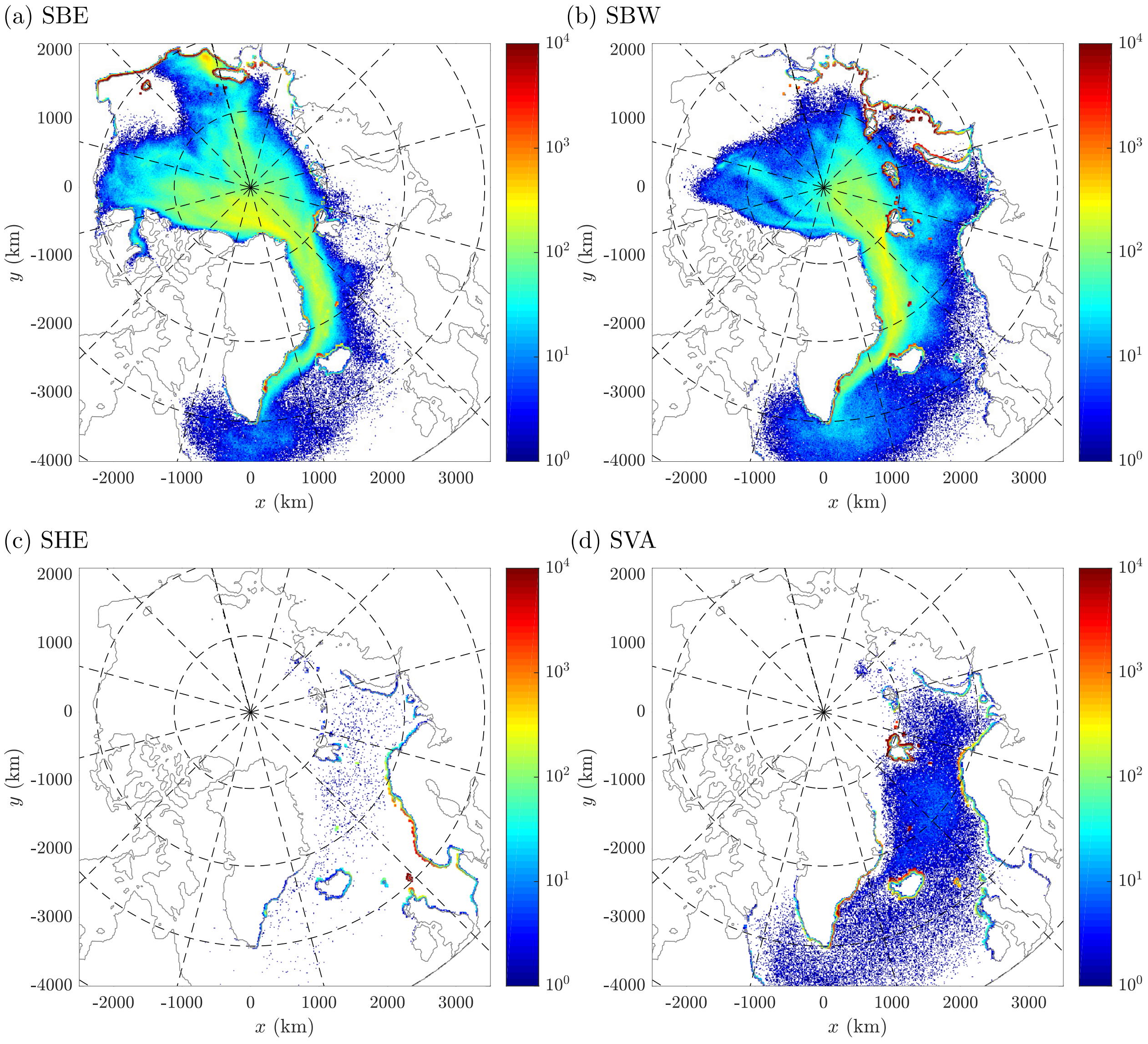

Supplementary Figure 22: As in Supplementary Fig. 9, but for particles released from SBE, SBW, SHE and SVA (a–d). (Maps created with Matlab version 2016b, `https://www.mathworks.com/`.)
